# Synthesis of the Evidence on What Works for Whom in Telemental Health: Rapid Realist Review

**DOI:** 10.2196/38239

**Published:** 2022-09-29

**Authors:** Merle Schlief, Katherine R K Saunders, Rebecca Appleton, Phoebe Barnett, Norha Vera San Juan, Una Foye, Rachel Rowan Olive, Karen Machin, Prisha Shah, Beverley Chipp, Natasha Lyons, Camilla Tamworth, Karen Persaud, Monika Badhan, Carrie-Ann Black, Jacqueline Sin, Simon Riches, Tom Graham, Jeremy Greening, Farida Pirani, Raza Griffiths, Tamar Jeynes, Rose McCabe, Brynmor Lloyd-Evans, Alan Simpson, Justin J Needle, Kylee Trevillion, Sonia Johnson

**Affiliations:** 1 NIHR Mental Health Policy Research Unit Division of Psychiatry University College London London United Kingdom; 2 NIHR Mental Health Policy Research Unit Institute of Psychiatry, Psychology and Neuroscience King’s College London London United Kingdom; 3 Centre for Outcomes Research and Effectiveness Research Department of Clinical, Educational and Health Psychology University College London London United Kingdom; 4 NIHR Mental Health Policy Research Unit Lived Experience Working Group Division of Psychiatry University College London London United Kingdom; 5 Camden and Islington NHS Foundation Trust London United Kingdom; 6 South London and Maudsley NHS Foundation Trust London United Kingdom; 7 Centre for Mental Health Research City University of London London United Kingdom; 8 Department of Psychology Institute of Psychiatry, Psychology & Neuroscience King's College London London United Kingdom; 9 Social, Genetic & Developmental Psychiatry Centre Institute of Psychiatry, Psychology & Neuroscience King's College London London United Kingdom; 10 Centre for Anxiety Disorders & Trauma South London & Maudsley NHS Foundation Trust London United Kingdom; 11 Psychological Medicine & Older Adult Directorate South London & Maudsley NHS Foundation Trust London United Kingdom; 12 Centre for Health Services Research City University of London London United Kingdom

**Keywords:** telemental health, remote care, telemedicine, mental health, COVID-19, digital exclusion, realist review, telemedicine, virtual care, rapid realist review, gray literature, therapy, health care staff, digital consultation, frontline staff, children, inpatient, mobile phone

## Abstract

**Background:**

Telemental health (delivering mental health care via video calls, telephone calls, or SMS text messages) is becoming increasingly widespread. Telemental health appears to be useful and effective in providing care to some service users in some settings, especially during an emergency restricting face-to-face contact, such as the COVID-19 pandemic. However, important limitations have been reported, and telemental health implementation risks the reinforcement of pre-existing inequalities in service provision. If it is to be widely incorporated into routine care, a clear understanding is needed of when and for whom it is an acceptable and effective approach and when face-to-face care is needed.

**Objective:**

This rapid realist review aims to develop a theory about which telemental health approaches work (or do not work), for whom, in which contexts, and through what mechanisms.

**Methods:**

Rapid realist reviewing involves synthesizing relevant evidence and stakeholder expertise to allow timely development of context-mechanism-outcome (CMO) configurations in areas where evidence is urgently needed to inform policy and practice. The CMO configurations encapsulate theories about what works for whom and by what mechanisms. Sources included eligible papers from 2 previous systematic reviews conducted by our team on telemental health; an updated search using the strategy from these reviews; a call for relevant evidence, including “gray literature,” to the public and key experts; and website searches of relevant voluntary and statutory organizations. CMO configurations formulated from these sources were iteratively refined, including through discussions with an expert reference group, including researchers with relevant lived experience and frontline clinicians, and consultation with experts focused on three priority groups: children and young people, users of inpatient and crisis care services, and digitally excluded groups.

**Results:**

A total of 108 scientific and gray literature sources were included. From our initial CMO configurations, we derived 30 overarching CMO configurations within four domains: connecting effectively; flexibility and personalization; safety, privacy, and confidentiality; and therapeutic quality and relationship. Reports and stakeholder input emphasized the importance of personal choice, privacy and safety, and therapeutic relationships in telemental health care. The review also identified particular service users likely to be disadvantaged by telemental health implementation and a need to ensure that face-to-face care of equivalent timeliness remains available. Mechanisms underlying the successful and unsuccessful application of telemental health are discussed.

**Conclusions:**

Service user choice, privacy and safety, the ability to connect effectively, and fostering strong therapeutic relationships need to be prioritized in delivering telemental health care. Guidelines and strategies coproduced with service users and frontline staff are needed to optimize telemental health implementation in real-world settings.

**Trial Registration:**

International Prospective Register of Systematic Reviews (PROSPERO); CRD42021260910; https://www.crd.york.ac.uk/prospero/display_record.php?ID=CRD42021260910

## Introduction

### Background

Telehealth is defined as “the delivery of health-related services and information via telecommunications technologies in the support of patient care, administrative activities, and health education” [1]. Telemental health refers to such approaches within mental health care settings. It can include care delivered by means such as SMS text messaging and chat functions but most commonly refers to telephone calls and video calls, which are central to telemental health care.

Before the COVID-19 pandemic, there was interest in many countries and settings in integrating new technologies, including telemental health approaches, more widely and effectively in mental health care services. This was of particular interest in countries where face-to-face (ie, in-person) mental health care was largely inaccessible to remote communities [2]. Research has demonstrated that telemental health can be successful in various contexts, although studies before the pandemic tended to relate to relatively small-scale and well-planned applications of telemental health with volunteer participants, rather than large-scale implementations across whole service systems. Telemental health has been found to be effective in reducing treatment gaps and improving access to mental health care for some service users [3-5]. This includes those who live far from services or where caring responsibilities affect their ability to travel [6-8]. Positive outcomes and experiences have been reported across a range of populations (including adult, child and adolescent, older people, and ethnic minority groups) and settings (including hospital, primary care, and community) [9-11]. Some evidence has suggested that telemental health modalities such as videoconferencing are equivalent to, or even better than, face-to-face modalities for some service users in terms of quality of care, reliability of clinical assessments, treatment outcomes, or adherence [9,10,12,13]. High levels of service user acceptance and satisfaction with telemental health services have also been reported in research samples [4] and for certain populations, including those with physical mobility difficulties, social anxiety, or severe anxiety disorders [6,7]. However, conversely, telemental health services are not appropriate for or favored by all service users, and there is no one-size-fits-all approach. In particular, service users experiencing social and economic disadvantages, cognitive difficulties, auditory or visual impairments, or severe mental health problems (such as psychosis) have benefited less from telemental health interventions [14,15]. Digitally excluded service users tend to be people who are already experiencing other forms of disadvantage and are already at risk of poorer access to services and less good quality care; thus, a switch to telemental health may exacerbate existing inequalities [16,17]. In addition, concerns have been raised around the impacts of telemental health on privacy and confidentiality of clinical contacts, especially for the many service users who do not have the appropriate space and facilities for its use, as well as its appropriateness for certain purposes, such as conducting assessments or risk management [14].

Encouraging evidence of telemental health acceptability and effectiveness from prepandemic research tended to relate to limited populations who had opted into well-planned remote services [18]. However, during the COVID-19 pandemic, the use of telemental health around the world greatly accelerated, and telemental health became a routine approach for maintaining and delivering mental health services. Telemental health initiatives were central to delivering mental health services in the context of this emergency. Technological initiatives have also helped to address social isolation, which worsened throughout the pandemic [6,19]. In the United Kingdom, there were large increases in remote consultations in National Health Service (NHS) primary care [20], and national data reported that most contacts in NHS mental health settings were delivered remotely in 2020 [21], particularly during the first UK lockdown (March to July 2020).

Following the rapid adoption of telemental health at the start of the crisis, service planners, clinicians, and service users have expressed interest in the greater use of telemental health in the long term [14,19,22,23]. However, several challenges have been identified as arising from this widespread implementation [14,16,19,24,25]. These include (1) reaching digitally excluded populations, who may, for example, have limited technological access or expertise or both, thus compounding existing inequalities experienced by disadvantaged groups; (2) a lack of staff competence in using telemental health devices and confidence in delivering telemental health care; (3) a lack of technological infrastructure within health services; (4) challenges in managing clinical and technological risks in remotely delivered care; (5) developing and maintaining strong therapeutic relationships online, especially when the first contact is remote rather than face-to-face; (6) maintaining service user safety and privacy; and (7) delivering high-quality mental health assessments without being able to see or speak to the service user face-to-face. It is also more difficult to undertake physical assessments, including for physical signs linked to mental health, and side effect monitoring.

Both for future emergency responses and to establish a basis for the integration of telemental health into routine service delivery (where appropriate) beyond the pandemic, evidence is needed on how to optimize telemental health care, given the unique relational challenges associated with mental health care, and identify what works best and for whom in telemental health care delivery and in which contexts. It is also important to identify contexts in which telemental health is unlikely to be safe and effective, where face-to-face delivery should remain the default.

A methodological approach developed to address questions of which interventions work, for whom, and in which contexts in a timely way is the rapid realist review (RRR) [26]. This methodology has been developed to rapidly produce policy-relevant and actionable recommendations through a synthesis of peer-reviewed evidence and stakeholder consultation. A key characteristic of realist methodology is the focus on interactions between contextual factors (eg, a certain population, geographical location, service setting, or situation) and relevant mechanisms (eg, behavioral reactions, participants’ reasoning, or resources), which affect the outcomes of interest, such as intervention adherence or service user satisfaction [26-28]. Together, these are used to develop context-mechanism-outcome (CMO) configurations, which comprise the fundamental building blocks of realist synthesis approaches. Evidence from the wider literature is also drawn upon to develop midrange theories. Midrange theories are program theories that aim to describe how certain mechanisms in specific contexts result in specific outcomes [29]; the use of the wider literature to develop midrange theories helps to elaborate and refine the developed CMO configurations by shedding further light on how their mechanisms operate [26,30-32]. Additional information on realist terminology can be found in Multimedia Appendix 1.

### Objective

This is a unique opportunity to establish the characteristics of high-quality telemental health services and use these findings to identify key mechanisms for acceptable, effective, and efficient integration of telemental health services into routine mental health care. Using a realist methodology, in this RRR, we aimed to answer the question of what telemental health approaches work, for whom, in which contexts, and how? Specifically, we investigated the following questions in this review:

1. What factors or interventions improve or reduce adoption, reach, quality, acceptability, or other relevant outcomes in the use of telemental health in any setting?
2. Which approaches to telemental health work best for which staff and service users in which contexts?
3. In what contexts are phone calls, video calls, or SMS text messages preferable, and in which contexts should mental health care be delivered face-to-face instead?

We focus particularly on groups and contexts identified as high priority by policy makers (the process is described in detail in the *Methods* section), including children and young people, crisis care and inpatient settings, and groups at high risk of digital exclusion; examples from these groups are included wherever possible.

## Methods

### Overview

The RRR was conducted by the National Institute for Health Research Mental Health Policy Research Unit (MHPRU), a team established to deliver evidence rapidly to inform policy making, especially by the Department of Health and Social Care in England, associated government departments, and NHS policy leadership bodies. The project constitutes the final stage in a program of work on telemental health delivery conducted to meet urgent policy needs, which included an umbrella review of pre–COVID-19 evidence [18], a qualitative investigation of service user experiences of telemental health [24], a systematic review of literature on telemental health adoption conducted during the early phase of the pandemic [14], and a systematic review on the cost-effectiveness of telemental health approaches (personal communication by Clark et al, 2022). This RRR was registered on PROSPERO (International Prospective Register of Systematic Reviews; CRD42021260910).

We conducted the RRR during the COVID-19 pandemic, with videoconferencing via Zoom (Zoom Video Communications) as the primary means of communication among the research team.

### Study Design

An expert reference group of 28 people, including 16 (57%) university-employed academics, 7 (25%) experts by experience (lived experience researchers from the MHPRU Lived Experience Working Group (LEWG) with personal experiences of using mental health services or supporting others or both), and 8 (29%) experts by profession (including frontline clinicians), guided and contributed to the RRR throughout. Some members belonged to multiple groups and therefore worked from several (academic, clinical, or lived experience or multiple) perspectives.

The group met weekly throughout this process from July to November 2021. The expert reference group meetings served to develop and refine the study protocol; plan the searches for evidence (particularly the targeted additional searches supplementing the initially planned strategy); iteratively examine, refine, and validate the CMO configurations derived from our evidence synthesis, with reference to their expertise by experience or profession or both; and plan wider consultation on our emerging findings. Members of the expert reference group also contributed to the literature searches, data extraction, synthesis, and interpretation of data.

The stages of our RRR were based on the following five steps, variations of which have been described and used in previous studies [26,31,32]:

1. Developing and refining research questions
2. Literature searching and retrieving information (data/stakeholder views)
3. Screening and extracting information/data
4. Synthesizing information/data
5. Interpreting information/data

Our approach to these steps was iterative rather than linear, particularly for steps 3, 4, and 5, where there were multiple phases of extraction, synthesis, and interpretation. This is described in detail in the following section.

### Developing and Refining the Research Question

We formulated the research question in response to policy maker needs. We reviewed findings from earlier stages of the MHPRU’s program on COVID-19 impact on mental health care and on telemental health [6,18,19,24,33] with policy makers, including senior officials and mental health teams in the Department for Health and Social Care, NHS England, and Public Health England. We then identified questions to be addressed from their perspective to plan for future implementation and delivery of telemental health. This included considering how best to incorporate telemental health in routine practice once the need for its emergency deployment passes. Early in these discussions, three priority groups regarding the evidence that is currently lacking were identified as especially important for policy and planning: children and young people, users of inpatient and crisis care services, and digitally excluded groups. Digitally excluded groups include those who have no or reduced access to the digital world because of a lack of digital skills (eg, using computers or smartphones), connectivity (eg, access to the internet or phone signal), and accessibility (eg, those who may require assistive technology or do not have access to digital devices or connectivity because of, eg, costs). Our primary question of which telemental health approaches work, for whom, and in what context originated in these discussions and was further refined by the MHPRU core research team who identified a RRR methodology as appropriate and further refined the primary and secondary questions and methodology with the expert reference group before registering the protocol.

### Selection Criteria

Sources were included if they met the criteria described in the following sections.

#### Participants

Participants included staff working in the field of mental health, people receiving care from mental health services, family members, and other supporters of people receiving mental health care.

#### Interventions

Interventions included any form of remote (spoken or written) communication between mental health professionals, or among mental health professionals and service users, family members, and other supporters, using video calls, telephone calls, SMS text messaging services, or hybrid approaches combining face-to-face and web-based modalities. Peer support communications were also included alongside any strategies or training programs to support the implementation of the abovementioned interventions. Self-guided web-based support and therapy programs were excluded.

#### Types of Evidence

This included qualitative or quantitative evidence on (1) what improves or reduces adoption, reach, quality, acceptability, or clinical outcomes in the use of telemental health; (2) impacts of introducing interventions or strategies intended to improve adoption, reach, quality, acceptability, or clinical outcomes; (3) interventions or strategies intended to help mental health staff make more effective use of telemental health technologies; (4) impacts of telemental health on specific service user groups and settings, including people who are digitally excluded, users of inpatient and crisis care services, and children and young people; and (5) the appropriateness of the use of telemental health versus face-to-face care in particular contexts. In addition to outcomes, sources were required to include information on mechanisms (ie, what works, for whom, and how).

#### Study Design

This included any qualitative, quantitative, or mixed methods study design, including relevant service evaluations, audits, and case series. Gray literature and other sources, such as websites, stakeholder feedback, and testimonies from provider organizations and service user and carer groups were also included. Sources were also included if the focus was not solely on remote working, but the results contained substantial data relevant to our research questions. Substantial data had to provide relevant and sufficient information on context, mechanisms, and outcomes and contribute to the development of overarching CMO configurations. Editorials, commentaries, letters, conference abstracts, and theoretical studies were excluded.

### Search Strategy

**Figure 1:**
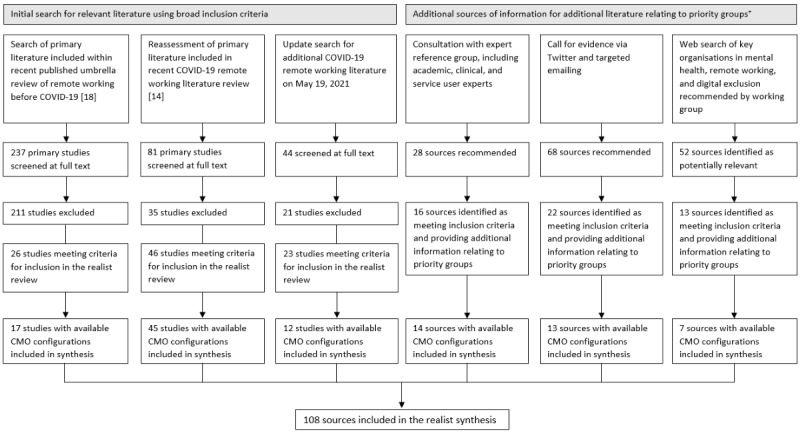
PRISMA (Preferred Reporting Items for Systematic Reviews and Meta-Analyses) diagram. *Inpatient and crisis service users, children and young people, people who are digitally excluded, and items from a service user viewpoint [14,18]. CMO: context-mechanism-outcome.

The search strategy was in accordance with PRISMA (Preferred Reporting Item for Systematic Reviews and Meta-Analyses) guidelines (Figure 1) [34]. Resources and literature were identified through the sources described in the following paragraphs.

First, we screened peer-reviewed studies included in 2 previous reviews on telemental health conducted by the MHPRU. The umbrella review by Barnett et al [18] included systematic reviews, realist reviews, and qualitative meta-syntheses on remote working before the onset of the COVID-19 pandemic [18]. The systematic review by Appleton et al [14] synthesized primary research on the adoption and impacts of telemental health approaches during the pandemic [14]. An updated search of the latter review was conducted on May 19, 2021*.*

Second, we worked with our expert reference group to identify additional peer-reviewed and gray literature. Searches were conducted on the websites of relevant national and international voluntary and statutory organizations identified by the expert reference group and by internet searches (eg, Mind and the Royal College of Psychiatrists). Identified literature was noted on a shared Microsoft Excel spreadsheet. This process was supported using Slack, a web-based messaging application, to coordinate this complex and rapidly changing process.

Finally, the MHPRU disseminated a call for evidence via Twitter and email to relevant organizations and individuals (such as charities supporting digital inclusion, chief information officers, and telehealth leads within NHS Trusts) inviting them to submit relevant evidence, including evaluations, audits, surveys, stakeholder feedback, and testimonies from provider organizations and service user and carer groups.

### Study Selection

References included in the umbrella and systematic review were downloaded and screened for inclusion in the RRR using EPPI-Reviewer (version 4.0) [35]. One of the reviewers screened abstracts and titles of the references identified through the updated searches of the umbrella review and systematic review. Full texts were reviewed for inclusion, with included and *unsure* sources checked by another reviewer. Sources were included in the final review if they met our inclusion criteria and provided relevant information for the development of CMO configurations. Disagreements were resolved through discussion with the wider research team.

### Process of Data Extraction, Synthesis, and Interpretation

#### Data Extraction

The source characteristics were extracted and inputted on EPPI-Reviewer (version 4.0) [35]. Extracted characteristics included study aim and design (if applicable), type of service, telemental health modalities used, mental health diagnosis of service users, and staff occupation. MHPRU researchers screened each included source for information that could be assembled into CMO configurations related to telemental health (ie, information on contexts, outcomes, and underlying mechanisms). Underlying CMO configurations were extracted by MHPRU researchers and LEWG members. Underlying CMO extraction involved reading each source before identifying contexts, mechanisms, and outcome configurations that were either (1) identified by the authors in the paper or (2) identified by extractors by linking them together from the data, descriptions, and discussion in the paper. Each week, samples of the extracted CMO configurations were reviewed by the expert reference group to ensure coherence, relevance, validity, and format consistency.

#### Data Synthesis

The research team then began the process of synthesizing the underlying CMO configurations by reviewing the extracted CMO configurations and identifying emerging themes. We developed four domains to encapsulate key aspects of the evidence: (1) connecting effectively; (2) flexibility and personalization; (3) safety, privacy, and confidentiality; and (4) therapeutic quality and relationship. Each of the 4 identified domains was allocated to an MHPRU researcher to lead the synthesis, with input from LEWG members, clinicians, and MHPRU senior researchers.

To develop content for each of these 4 domains, underlying CMO configurations were, in essence, synthesized based on similarities to create a single overarching CMO (discussed in full detail in Multimedia Appendix 1). Underlying CMO configurations extracted from individual sources were reviewed in terms of their similarities and differences (eg, CMO configurations related to the convenience of telemental health) and then grouped together based on similar mechanisms (eg, flexibility, which reduces practical barriers to accessing mental health care) and outcomes (eg, increasing attendance and reducing missed appointments). Each similar group of underlying CMO configurations was then synthesized and refined to create a single overarching CMO, which reflected key content across the underlying CMO configurations, as well as input from the expert reference group. Each overarching CMO was assigned to 1 of the 4 domains.

Realist work does not conduct a traditional quality appraisal, as it values evidence from all sources in a nonhierarchical manner [27,29]. Our overarching CMO configurations were also significantly developed throughout the synthesis process based on stakeholder input, and thus, quality appraisal of individual study sources would not have reflected the quality of the final overarching CMO configurations.

#### Interpretation

An iterative process of revising and refining overarching CMO configurations from the perspective of stakeholder experience followed. Revisions, refinements, and additions were first made through discussion with the expert reference group. Summaries were then also discussed at three 2-hour stakeholder webinars, each focusing on one of our priority groups: children and young people, inpatient and crisis care services, and digitally excluded groups. The webinars were primarily attended by groups representing these constituencies and services who work with them, including experts and stakeholder representatives from research, policy, and clinical settings (nationally and internationally); the voluntary sector; lived experience groups and community organizations working with marginalized groups; and telehealth technology initiatives. There were between 30 and 40 participants at each webinar. During the webinars, participants were divided into breakout rooms, with a facilitator and a note taker from the core research team. High-level summaries of preliminary data were presented by domain, and attendees were asked to discuss the following questions: (1) whether the preliminary summaries captured their own knowledge and experience of telemental health, (2) whether the summaries applied to and how the summaries were relevant for the priority group at hand, and whether they were aware of any additional challenges or recommendations related to delivering telemental health to the priority group.

On the basis of the feedback from these webinars, the overarching CMO configurations within each of the 4 domains were then further revised and refined. We actively sought additional information related to each overarching CMO, including relevant contexts, further detail about mechanisms, real-life examples of strategies and solutions (such as for overcoming barriers identified within the CMO), and points of particular importance or concern, from the webinar notes, the expert reference group meetings, and related literature. We noted this information alongside the relevant overarching CMO and used it to refine the CMO configurations. In addition, we drew upon midrange theories (evidence-based theories derived from the wider literature) to provide more theoretically informed explanations of mechanisms (eg, the digital inverse care law [16], which theorizes that those most in need of care via telemental health are least likely to engage with it and existing inequalities will widen, helped to strengthen the mechanisms around digital exclusion). Throughout this process, the core research team and the expert reference group were iteratively consulted, and their feedback was integrated into the overarching CMO configurations. The revised theories were shared for a final email consultation with the stakeholders who were invited to our webinars. Their feedback was incorporated and resulted in the final overarching CMO models presented under each domain in this paper.

## Results

### Overview

Underlying CMO configurations were extracted from 17 studies included in the previous umbrella review [18] and from 45 studies included in the systematic review [14]. The updated search yielded 44 potentially relevant studies, of which 21 (48%) were excluded (either as they did not contain data on context, mechanisms, and outcomes or they added no additional information as data saturation had been reached). CMO configurations were extracted from 52% (12/23) of the remaining studies that met our inclusion criteria and were included in the realist synthesis. Through consultations with our expert reference group, we identified 28 sources, of which 16 (57%) met our inclusion criteria and provided additional information related to our priority groups, and 14 (50%) yielded CMO configurations that were included in the synthesis. We received 68 potential sources through the call for evidence, of which 22 (32%) met our inclusion criteria and provided relevant information on our priority groups; CMO configurations were extracted from 13 (19%) of these. Finally, website searches identified 52 potentially relevant sources, of which 13 (25%) met our inclusion criteria and 7 (13%) provided information relevant to CMO configurations. The realist synthesis includes a total of 108 sources.

Of the 108 included sources with primary data or detailed accounts of what works, for whom, and in what context, most were primary research studies (72/108, 66.7%), followed by service descriptions/evaluations/audits (19/108, 17.6%), guidance documents (4/108, 3.7%) and briefing papers (3/108, 2.8%), commentaries/editorials/discussions (4/108, 3.7%), and letters (2/108, 1.9%), as well as a review (1/108, 0.9%), news article (1/108, 0.9%), webpage (1/108, 0.9%), and a service user–led report (1/108, 0.9%). Of the 84 sources that included primary research data, 32 (38%) used quantitative methods, 19 (23%) used qualitative methods, and 33 (39%) used mixed methods (including n=2, 2% case studies).

Most sources were published in the United States (41/108, 38%) and the United Kingdom (34/108, 31.5%). The remaining sources collected data in Canada (7/108, 6.5%), the Dominican Republic (1/108, 0.9%), Australia (7/108, 6.5%), China (2/108, 1.9%), India (3/108, 2.8%), Egypt (1/108, 0.9%), and Nigeria (1/108, 0.9%), as well as 10 European countries, including Austria (1/108, 0.9%), France (1/108, 0.9%), Germany (1/108, 0.9%), Ireland (1/108, 0.9%), Italy (2/108, 1.9%), Netherlands (1/108, 0.9%), Portugal (1/108, 0.9%), Spain (1/108, 0.9%), Sweden (1/108, 0.9%), and Switzerland (1/108, 0.9%). Details of the included sources are presented in Multimedia Appendix 2 [12,19,24,36-153].

Overarching CMO configurations for each domain are summarized in Tables 1-4, and details of the underlying CMO configurations and summary notes on stakeholder discussions, which shaped the overarching CMO configurations, are presented in Multimedia Appendix 3 [12,19,24,36-153]. For each overarching CMO, we included examples of key contexts that are relevant for the CMO and examples of strategies and solutions addressing the challenges or opportunities identified in the CMO: these were drawn from underlying CMO configurations and stakeholder discussions. In the text outlining each domain, we also identify major midrange theories that elucidate mechanisms and outcomes for overarching CMO configurations.

### Domain 1: Connecting Effectively

The content of this domain relates to establishing a good web-based connection to join a video call of sufficient quality or to engage in telemental health via phone or message, with a particular focus on digital exclusion. Table 1 outlines 7 overarching CMO configurations identified in relation to this domain, addressing issues concerning device and internet access (CMO 1.1 and CMO 1.2), technology training (CMO 1.3), the impact of preparation and technological disruptions (CMO 1.4 and CMO 1.5), the familiarity and usability of the platforms (CMO 1.6), and the acceptability of telemental health as an alternative to receiving no care during emergency situations (CMO 1.7). Three of the CMO configurations were related to trying to resolve three main challenges: (1) access to a charged, up-to-date device that enables internet access (CMO 1.1); (2) an internet (Wi-Fi or data) or signal connection (CMO 1.2); and (3) the knowledge, ability, and confidence to engage on the web (CMO 1.3). Much of the content relates to challenges service users encounter in engaging with telemental health; however, the literature and stakeholder discussion also yielded significant challenges for staff and service providers in the practicalities of connecting on the web.

Theories regarding the relationship between digital exclusion and other forms of exclusion and deprivation, as well as the potential of digital exclusion to amplify inequalities, contribute to our understanding of key mechanisms and outcomes in this domain. Widening inequalities have been described as an inevitable consequence of the expansion of the role of technology in health care, with loss of access to community facilities, such as libraries, making this a still greater risk during the pandemic [154]. The “digital inverse care law” [16] describes a tendency for groups in most need of care (eg, older people or people experiencing social deprivation) to be least likely to engage with technological forms of health care. This is highly salient in mental health care, given the strong associations between experiencing mental health challenges and experiencing one or, often, many forms of disadvantage [154].

Access to devices (CMO 1.1) is a contributor to digital exclusion, and groups who are especially likely to be affected include homeless individuals and people living in poverty, those receiving inpatient or crisis care, and young children who may not have their own devices. The type of device may be important for accessing telemental health. For example, smartphones may be less suitable for video therapy because of their small screens [155], although this may be less relevant for young people who are familiar with and consistently use smartphones for connecting on the web [156]. This raises future research questions around which types of digital devices work for whom and in what context when it comes to continuing telemental health treatment. It may also have implications for the provision of suitable equipment to certain populations.

Our consultations and the wider literature revealed that lack of access to good quality Wi-Fi, including poor Wi-Fi in hospitals and offices, was a further key barrier to the successful and equitable delivery of telemental health (CMO 1.2). It was emphasized that modernization of software and hardware, particularly within the NHS, is needed in many health care sites to allow for the requirements of telemental health. Service users also reported relying on their own mobile phone data to connect to telemental health services, often depleting their data completely after or during just one video call or consultation, which is expensive to replenish and may also deter engagement. This could amplify existing inequalities, leaving some service users at risk of digital exclusion and unable to access the internet and mental health support. Disruptions to telemental health appointments because of poor connections are a significant barrier to engagement (CMO 1.4). Our consultations and the existing literature highlighted the importance of having an alternate form of communication (eg, a telephone call) as a backup plan in case of a technology or connection failure [36,37,157,158].

**Table 1:** Domain 1: Connecting Effectively.

CMO^a^ title		References	Overarching CMO	Key contexts	Example strategies and solutions
CMO 1.1: Providing *service users* with access to digital devices		[19,39-49, 59]	When service users who do not have access to digital devices are given access to up-to-date devices (and chargers), paid for or loaned to them (context), this results in improved access to and implementation of telemental health services (especially via video platforms; outcome 1), some inequalities in accessing a digital device are addressed (outcome 2), exacerbation of existing inequalities is less likely (outcome 3), and service users are more able to maintain personal contact with family and friends if they wish and access a range of web-based services (outcome 4), as this reduces the burden of having to purchase a device for the service users and provides more financially viable access to devices required for web-based connections (mechanism).	Lack of access to devices particularly affects people living in poverty or unstable living circumstances (such as homeless people or refugees), as well as other groups at risk of exclusion not only from telemental health but from a range of services and networks (such as people who are cognitively impaired or with psychosis or substance abuse disorders). It can also particularly affect inpatients, who may not have access to devices or charging facilities or both on the ward, and children and young people, who may not have access to their own devices.	Schemes organized by bodies, including health care providers, libraries, schools and colleges, charities, and community organizations, that lend or give digital devices and chargers Inpatient wards providing devices, such as iPads, and short cable or wireless chargers, or charging lockers Health services providing separate and private rooms with videoconferencing capabilities Promoting awareness among service providers about the tendency for digital exclusion to exacerbate existing disadvantages and inequalities, with the development of active strategies to mitigate this Phone calls rather than video calls may be more appropriate for service users who do not have access to digital devices that facilitate video calls
**CMO 1.2: Lack of access to stable, secure, and adequate internet connection**					
	Staff	[19, 39, 41, 42, 44, 47, 50-60]	When the staff deliver telemental health via video from workplaces or homes with an unstable and poor internet connection (context), teleconsultations are difficult (or impossible) to conduct with service users (outcome 1), fewer teleconsultations are conducted (outcome 2), and telemental health is viewed less positively (outcome 3), as staff experience frustration, and there is reduced motivation to arrange web-based appointments (mechanism).	This is particularly relevant for staff working within health care providers that frequently have insufficient Wi-Fi or internet connection to deliver sessions smoothly. It also affects staff who do not have adequate Wi-Fi connectivity in their own homes or when working in the community.	Investing in high-quality IT infrastructure to ensure disruptions to calls do not originate from poor provider connections Providing devices with access to data, or data/Wi-Fi allowances, for use by staff when working away from health service premises (at home or in community settings)
	Service users	[19, 39, 41, 42, 44, 47, 50-60]	When service users only have access to an insecure, unstable, and poor-quality internet connection or consistent technological problems (context), it is difficult for telemental health to be viewed positively (outcome 1), they are able or willing to accept fewer web-based consultations (outcome 2), they may continue to struggle with their mental health (if face-to-face consultations are unavailable; outcome 3), and this may result in digital exclusion that could exacerbate existing inequalities (outcome 4), as the service users struggle to engage in sessions with sufficient clarity and mutual comprehension and experience frustration (mechanism).	This is especially relevant for service users on low incomes or from socially marginalized groups (such as homeless people), those living in multiple occupancy households where Wi-Fi is overstretched, and people from low-to-middle income countries. Lack of access to reliable internet or even electricity also differentially affects people in rural and remote areas. People in marginalized groups may also lack the means to pay for a telephone service.	Signposting to low-cost plans for people on low incomes Providing free access to Wi-Fi, data, and phone connections (may be included with devices that are lent or given) Providing or signposting to community hubs with access to data Providing face-to-face appointments where the above problems cannot be resolved
**CMO 1.3: Benefits of providing support and guidance for using technology**					
	Staff	[19, 24, 38, 42-44, 46, 53, 59, 61-75]	When staff who lack the confidence or knowledge to deliver mental health care online (particularly via video calls) receive practical instruction and guidance on how to use technology to deliver mental health services (including clear information about how to operate within local policies, procedures, and platforms; troubleshoot issues during telemental health sessions; and formulate and implement backup plans; context), they feel an increased sense of confidence in managing and delivering telemental health services (mechanism), which leads to increased use of telemental health services (outcome 1) and fewer delays, resulting in more appointments being completed on time (outcome 2).	This is especially relevant for staff who are new to delivering mental health care remotely or who are unclear or unfamiliar with using locally recommended platforms and procedures.	Provision of training sessions relevant to the local context, including information on troubleshooting technology and maintaining privacy, safety, and confidentiality Access to guidance on video calls, including clear information on processes and policies for providing telemental health in the organizations where the staff work Peer support and group or team sessions for practicing technology Refresher training sessions and rolling training for new joining staff
	Service users	[19, 24, 38, 42-44, 46, 53, 61-75]	When service users with access to a technology device who struggle with the confidence, knowledge, or the ability to use telemental health receive guidance, reassurance, and instruction (tailored to their health care provider and their language, reading ability, and any sensory disability) on how to use technology (particularly video calls) to access mental health care, engage with backup plans, and receive timely technical support and troubleshooting during treatment sessions (context), they feel an increased sense of confidence in accessing telemental health (mechanism), which reduces anxiety in using telemental health and digital technologies (including in their personal lives; outcome 1), facilitates the adoption of and adherence to telemental health (outcome 2), improves service users’ ability to adjust to remote care (outcome 3), reduces interruptions in care delivered via telemental health (outcome 4), and increases satisfaction with telemental health (outcome 5).	Receiving guidance tailored to local policies and procedures for telemental health access is relevant to all service users. It is likely to be particularly relevant to groups identified as at high risk of digital exclusion through lack of confidence in using technology, including older people, people with severe mental health problems, and people with intellectual disabilities or cognitive impairments. Production of accessible guidance is especially relevant for people with cognitive impairments or intellectual disabilities, children, people with sensory disabilities, and people who do not understand English well.	One-to-one training or support from a digital facilitator/champion/mentor, peer supporters, family, friends, or a dedicated member of staff who can provide guidance Written or video information about how to access telemental health, including material tailored to age, cognitive abilities, sensory impairments, and language, and to the platforms that are in local use (eg, guidance in other languages, easy-to-read material with pictures, and personalized workbooks sent to children before a telemental health contact) Opportunities for practice and repetition of training as needed Written guides for different technology platforms that give step-by-step instructions, including how to set up IDs and passwords, sent out with appointment letters Brief and direct training sessions offered to service users before their web-based appointment, performing a trial run of using the technology beforehand or increasing web-based contact duration to accommodate learning
CMO 1.4: Impact of technology-related disruptions		[36, 45, 56, 76-82]	When technological issues (including connection problems and device issues) lead to disruptions in web-based sessions and there is no prearranged backup method of contact (eg, a plan to connect by telephone instead of video call if needed; context), the quality of the intervention is diminished (outcome 1), there is a loss of empathic connection between client and therapist (outcome 2), and the sessions may not be able to continue (outcome 3), as the flow of the conversation is interrupted and session time reduced, for example, when having to ask the other person to repeat what has been said or when cut off completely, leaving staff and service users potentially feeling distracted, frustrated, awkward, and upset (particularly if there is a threat of therapy withdrawal because of missed sessions; mechanism).	This is particularly relevant for the use of videoconferencing, where phone backup can reduce the risk of abandoning appointments because of failure to make a stable connection.	Agree to a backup method of connecting (eg, reverting to telephone calls in the event of a disruption) Where connection issues cannot be readily resolved, and backup methods are not sufficient, strategies to address connection problems (as mentioned previously) are required, or moves to face-to-face care should be facilitated
CMO 1.5: Preparing *service users* for telemental health		[83-85]	When staff prepare service users for telemental health appointments and communicate clearly with service users about what to expect (context), this leads to more accepted calls and fewer missed service user contacts (outcome), as service users have more relevant knowledge of the process, including when to expect contacts and from what number, and feel more comfortable engaging with telemental health services (mechanism).	This is relevant across telemental health contexts but may be particularly relevant for service users who have not used telemental health before or are anxious or worried about telemental health.	Informing service users when they are going to be contacted and via what platform Sending texts to remind a service user that a call is scheduled for a specific time Informing service users about when phones are manned and how long they can expect to wait for a call back Routing staff telemental health calls to service users through unblocked, local, and familiar clinic or hospital numbers or, if a private number must be used, ensuring that service users are expecting this
CMO 1.6: *Service users’* familiarity with the platform and ease of use		[41,66,86-90]	Where service users already use remote technologies for social, educational, or work purposes, or where the web-based platforms are relatively easy to use (context), offering a choice of familiar and accessible technology platforms that may be less difficult and time-consuming for staff and service users to understand or learn (mechanism) may increase the likelihood of engagement with services via telemental health, especially video calls (outcome).	This is especially relevant to service users who are already making some use of technology, for example, for social, educational, or work purposes.	Service providers prioritizing allowing the use of a variety of platforms, especially those likely to be widely familiar (eg, Zoom and WhatsApp video) Attempting to address governance concerns and provide guidance for safe use, balancing web-based safety and other risks such as disengagement from services
CMO 1.7: Telemental health may be a better alternative to receiving no care for *service users* during an emergency, such as the COVID-19 pandemic		[36, 43, 60, 90-92]	When service users are offered telemental health appointments as face-to-face appointments are restricted (eg, because of COVID-19 or another emergency; context); these appointments are likely to be accepted by some service users on the basis that they are the main way by which mental health care can continue (outcome 1), and there is a reduced risk of infection from COVID-19 (outcome 2), as telemental health is seen as preferable to receiving no support at all and as an alternative to canceling appointments entirely (mechanism).	This is applicable across mental health services in the context of any emergency which restricts face-to-face meetings, especially in services where all face-to-face contacts have been discontinued or where they are limited to immediate crises. It is especially relevant for service users who need to self-isolate because of high personal risk, or where clinicians need to self-isolate after contact with others.	Widespread implementation of telemental health has proved a successful strategy for maintaining contact with many (but not all) mental health service users during an emergency that restricts face-to-face contact, with some service users accepting telemental health in this situation, but who would otherwise be reluctant or unable to do so if not in a relatively short-term emergency Some continuing face-to-face contacts are still required if care is to be offered to all who need it

^a^CMO: context-mechanism-outcome.

We identified the importance of technology training and sustained formal and informal support for service users (CMO 1.3). Variations in the ability to use telemental health are likely to disproportionately affect certain service user populations, often groups who also experience high levels of need for mental health care and inequalities in its provision. This includes people living in deprived circumstances, people with cognitive difficulties, people with paranoia, or those who do not speak the same language as service providers. Understanding how to use technology is also important for service users’ social engagement and connection, which is relevant for wider recovery and citizenship [159-161]. Young children may also be disproportionately affected as they may not be able to resolve difficulties they experience during telemental health sessions without the help of their parents or other supporters.

For staff (CMO 1.3), our evidence suggests that staff training provided more widely and accessibly on using technology for telehealth would be helpful to ensure high-quality service provision and overcome barriers around staff not having time allocated to training or being reluctant to ask for support. Evidence suggests that there are also benefits of having access to technical support to troubleshoot issues during sessions [38], as well as of practicing new skills and learning with colleagues and peers [162].

The importance of the familiarity and usability of platforms was highlighted throughout our stakeholder consultations and weekly reference group meetings, as well as in the published literature (CMO 1.6), in keeping with previous research on the acceptability of telemedicine by service users [163]. Many of the platforms and devices commonly used for telemental health services, for example, during the COVID-19 pandemic, were not designed for use in health care settings and, therefore, may be less user-friendly. The importance of usability is emphasized by Nielsen and Landauer [164]; 3 of 5 main usability attributes of a program are that it should be easy to learn, efficient to use, and easy to remember. This is in keeping with our findings related to familiarity and usability in telemental health.

Finally, preparing service users for telemental health sessions was key (CMO 1.5). This was relevant across telemental health contexts; however, information tailored to individual communication needs may be especially helpful for service users who may experience additional challenges connecting on the web (eg, those who are inexperienced with technology or anxious about using telemental health, young children, older people, and people with cognitive difficulties). For people with significant sensory impairments, specialist adaptations will need to be available if telemental health is to be a viable modality (eg, mobile phones that flash when receiving a call or providing guidance in Braille or sign languages).

### Domain 2: Flexibility and Personalization

Table 2 presents the CMO configurations, key contexts, and example strategies and solutions for the domain of flexibility and personalization. The need for flexibility and personalization was a key theme identified in both the literature and stakeholder consultations when considering using telemental health in place of (or in conjunction with) face-to-face mental health support. A total of eight overarching CMO configurations were identified in this domain, which can be divided into three main categories: taking individual preferences into account (CMO 2.1, CMO 2.5, and CMO 2.7), convenience (CMO 2.2), and allowing for more collaborative and potentially specialized care (eg, involving specialists, family, or friends in care; CMO 2.3, CMO 2.4, CMO 2.6, and CMO 2.8).

Our findings emphasize the importance of taking individual service user preferences into account when deciding whether to make use of telemental health, in selecting the modality of digital communication used (including the type of technology platform), and in decisions about involving others (clinicians or family members) in care. This finding underpins all other CMO configurations in this theme and coheres with theories regarding the importance of shared decision-making, collaborative care planning, and personalization in mental health care [165-167]. Involving service users and carers in decisions and care planning as part of a collaborative approach to mental health care has been identified as central to best practice [168]; for example, a review of collaborative care for depression and anxiety found this approach to be more effective than usual care in improving treatment outcomes [169].

Flexible use of telemental health was also identified as being beneficial in reducing barriers to accessing mental health support for some service users, particularly those who may struggle to access face-to-face services for reasons such as caring or work commitments, problems traveling (eg, because of a physical disability, anxiety, or lack of transport) or a reluctance to attend the stigmatizing places. Telemental health can also facilitate connections between clinicians, especially across different services or specialties, which can improve multidisciplinary working and collaboration across teams and agencies and provide service users with a wider range of specialists or support for specific groups. This approach has been identified as having salience in a mental health setting [170-172]. In some cases, telemental health was viewed by both service users and clinicians as more convenient, as it reduced the need for (and cost of) traveling to face-to-face appointments.

Telemental health was also seen as an important tool in inpatient wards, especially during the COVID-19 pandemic when visiting was restricted, as it allowed service users to stay in touch with family and friends and for them to be involved in their care. It also allowed staff supporting inpatients in the community to remain involved.

However, instances were identified where telemental health was not appropriate, and face-to-face care needs to be available. For example, some service users do not wish or feel able to receive care by remote means or do not wish to have all appointments by this means, whereas others may struggle with telemental health because of sensory or psychological factors or a lack of access to appropriate technology and internet connectivity.

**Table 2:** Domain 2: Flexibility and personalization.

CMO^a^ title	References	Overarching CMO	Key contexts	Example strategies and solutions
CMO 2.1: Taking *service users’* individual preferences into account—offering alternatives	[24, 36, 48, 50, 65, 67, 75, 77, 90, 91, 93-104, Eagle et al (email, August 31, 2022)]	When services using remote mental health care allow service users to choose the modality of telemental health and a choice of remote versus face-to-face care and regularly check their preferences (context), this allows service users to have greater autonomy and choice (mechanism), leading to them feeling more satisfied and able to engage with the type of care received (outcome 1), leading to improved uptake (outcome 2) and improved therapeutic relationships with their clinician (outcome 3).	Allowing service user choice and delivering services flexibly is a key principle across settings and populations, with the overall aim that care of equivalent quality should be available in a timely way whatever modality is chosen. Hybrid care, with a flexible mixture of face-to-face and telemental health care based on the purpose or function of appointment (eg, prescription review versus the first visit to see a clinician), preference, and circumstances, is especially relevant to service users receiving relatively complex care with multiple types of appointments, for example, from multidisciplinary community teams. Children and young people may particularly benefit from being offered a choice as it increases their feelings of autonomy and improves engagement in care.	Initial conversations about telemental health with all service users, in which their preferences regarding the mode of appointments and their access to and expertise and interest in using technology are explored (a shared decision-making tool could be used to structure this) Ensuring that clinicians making collaborative plans with service users for telemental health use are aware of risk factors for difficulties engaging with telemental health and digital exclusion, including individual difficulties and wider contextual factors, such as poverty and poor or shared housing Ensuring equal access to timely care of good quality regardless of choice of modality Regularly revisiting preferences and collaboratively planning how care will be delivered Ensuring that service users engaging in group therapies and activities have understood and consented to the ways of working of the group and that face-to-face alternatives are of equivalent quality
CMO 2.2: Removing barriers—greater convenience for *service users and family/friends*	[19, 24, 36, 38, 40, 42, 45, 54, 55, 67, 70, 75, 76, 79, 81, 82, 84, 90-92, 95, 97, 105-112]	Among some service users, family, and other supporters experiencing specific practical barriers to attending face-to-face services (childcare or other caring responsibilities; location, work, and mobility limitations; travel difficulties/costs, and work commitments) and those who have good access to telemental health (context), telemental health may provide increased flexibility that addresses individual practical barriers (mechanism), which can lead to telemental health being viewed by some service users and carers as more convenient and accessible than face-to-face care (outcome 1), easing attendance (outcome 2), increasing uptake (outcome 3), and reducing missed appointments (outcome 4).	This may be relevant for parents with young children, people with caring responsibilities, and people who struggle to travel because of work commitments/disability/costs; children and young people in school or higher education (so they can access mental health care without having to leave their place of education); people who live in remote areas or a long distance away from a specialist service; and people for whom travel is challenging because of impaired mobility or sensory impairments or mental health difficulties such as agoraphobia. There may be more advantages to treatments that involve the support of family and friends.	Offering explicit choice wherever possible between telemental health and face-to-face care, including home visits where services are able to provide this, also considering that different modalities may be used for different purposes Identification of people for whom attendance at office appointments is challenging so that telemental health (or home visits) can be considered Continuing to offer choice and checking preferences throughout the duration of care (ie, not just asking once) Avoiding missed appointments by offering a switch to telemental health as an option when a service user is unable at short notice to attend a face-to-face appointment
CMO 2.3: Involvement and support for *family and friends*	[79, 91, 113-115]	When family and other supporters are invited (with service user agreement) to join telemental health sessions (context), this may result in more holistic treatment planning and greater engagement of family and others in supporting service users (outcome 1); may help improve therapeutic relationships and treatment success (outcome 2), increase engagement (outcome 3), and reduce some uncertainty and anxiety around treatment (outcome 4); and may increase the satisfaction of and support for family and friends (outcome 5), as family and other supporters may be able to participate in care planning meetings and assessments that they would have found difficult to attend face-to-face, increasing their engagement in supporting service users and their understanding of their difficulties and care plans (mechanism).	This is especially helpful for those living in locations different from their family and friends or where family and friends have caring or work commitments preventing them from attending meetings face-to-face, children and young people (as this may allow their parents to be more involved in their care), and service users in inpatient settings where family and friends cannot visit (eg, because of epidemic-related restrictions) or as the hospital is in a remote location.	Working with service users to identify any family and friends whose attendance at care planning and other clinical meetings (including on inpatient wards) would be helpful, including those for whom telemental health would facilitate access, such as people in distant locations or whose commitments would make it difficult to attend face-to-face meetings Using strategies for service users to provide guidance on using telemental health to family and friends and prepare them for appointments Offering children and their families the opportunity to have telemental health appointments (or, if feasible, home visits) if they find it easier to participate as a family without having to travel to an appointment and to be seen in a clinical setting In inpatient wards, providing charged iPads, short cables, or charging lockers to allow service users to charge their own devices so that they can use technology to connect with family or other supporters
CMO 2.4: Widening the range of available mental health services and treatments for *service users* via telemental health	[49, 116-118]	For service users who may benefit from services that they cannot readily access locally and that provide specialized forms of treatment and support regionally or nationally (context), telemental health can widen the range of specialist assessment, treatment, and support available (mechanism), which potentially leads to improved access to services tailored to individual needs and culturally appropriate or specialist services (outcome 1) and improved satisfaction and treatment outcomes (outcome 2), although an impoverished range of local face-to-face provision may be a risk if referral to distant specialist care via telemental health becomes routine (outcome 3).	People to whom this is relevant may include people who have complex clinical needs or rarer conditions such that they would potentially benefit from assessment, treatment, and support from specialist services provided at regional and national rather than local levels; people who may be able to access distant therapists who speak their own language or interpreters of rare languages not available locally; people who would benefit from support from voluntary organizations that meet specific needs that are not catered for locally (eg, that support particular cultural groups; lesbian, gay, bisexual, transgender, and queer groups; or people with sensory impairments); and people who would benefit from a wider choice of therapies and support (including peer support) than is available locally.	Development (including of funding arrangements) and dissemination of information about specialist services accessible via telemental health Access for service users, their family and friends, and clinicians to information and signposting regarding community and voluntary sector organizations beyond their catchment area, which are accessible via telemental health Development of safeguards against the erosion of local and in-person national specialist services in favor of routine specialist telemental health, in line with public-sector equality duty to anticipate and provide for the needs of groups with protected characteristics under the Equality Act (eg, pregnant people who are at increased risk of domestic violence, people whose disabilities cause sensory hypersensitivity, and people who struggle with screen time)
CMO 2.5: Adaptations for *service users* with sensory or psychological barriers to telemental health	[40, 50, 77, 119]	Offering face-to-face (or telephone) appointments to people who struggle to cope with sensory (visual or auditory) aspects of telemental health or have symptoms that are exacerbated by it (context) may help to improve engagement with mental health care (outcome) as the adverse effects of the switch to telemental health for these symptoms and sensory or cognitive impairments may be avoided and service users are able to access their preferred modality of care (mechanism).	This may be relevant for people with symptoms that may interfere with or be exacerbated by engaging with telemental health, such as persecutory ideas or hearing voices; autism; sensory or cognitive impairments; and migraines.	Ensuring that face-to-face appointments (including home visits if there are impediments to office appointments) remain available Making clinicians aware of the types of clients who may find it particularly difficult to engage with telemental health Adapting telemental health where helpful, for example, through switching off cameras, using telephone rather than video calls, or communicating via SMS text message
CMO 2.6: Inclusion of multidisciplinary and interagency teams in *service users’* care	[89,115]	When mental health consultations are conducted using telemental health (context), it enables the inclusion of staff in appointments who are based geographically far away or who have schedules that would not have allowed them to join a face-to-face session (outcome 1), meaning care and support has potential to be more holistic and integrated (outcome 2), as it is possible for staff from different services and sectors to provide perspectives and contribute to plans (mechanism).	Key contexts include hospital inpatients, where telemental health may enable staff who work with them in community settings to join reviews and ward rounds (especially in pandemic conditions where they cannot attend in person), and people with complex treatment and support, who are receiving support from >1 team or sector.	Working with service users to identify staff whom it would be helpful to involve in consultations such as review and care planning meetings, including in social care, housing, and the voluntary sector Facilitating the involvement of such staff in reviews via telemental health, especially where face-to-face attendance is not feasible
CMO 2.7: Continuing to offer face-to-face care to *service users*	[53, 81, 92, 116, 120]	When service providers offer care of equivalent quality and timeliness face-to-face (including home visits where needed) rather than via telemental health to service users who do not wish or do not feel able to receive their care remotely (context), it ensures that care can continue and that inequalities in provision are not created or exacerbated (outcome), as it provides a choice to service users and avoids the negative impacts of digital exclusion (mechanism).	People for whom face-to-face options may be preferable, and choice is especially important, include those who do not have access to private spaces, live with people they do not wish to be overheard by in their appointments (including perpetrators of domestic abuse), do not feel comfortable communicating via remote means, and do not want therapy to intrude on their private lives should be included. In addition, some service users who value the time spent traveling to and from face-to-face appointments to process emotions may find face-to-face options particularly useful.	Ensuring services are able to offer a choice between telemental health and equivalent care delivered face-to-face (especially when telemental health is part of routine care rather than a means of managing a national emergency) Ensuring (as in CMO 2.1) that clinicians are fully aware of service user preferences and circumstances (which may be elicited via a shared decision-making tool) and continue to monitor these over time That clinicians are alert for any changing circumstances during telemental health where a service user does not feel comfortable to speak and make alternative arrangements accordingly (eg, using text functions on videoconferencing platforms or arranging face-to-face appointments)
CMO 2.8: Communication between *staff*	[19, 85, 121]	When remote technology platforms are used to facilitate real-time communication between staff members, including managers or clinicians working in different teams (context), it can lead to improved efficiency, more convenient working and staff management (outcome 1), improved communication and collaborative planning (outcome 2), and process improvement opportunities (outcome 3), as staff have the ability to rapidly share information, keep track of evolving telemental health procedures (eg, during emergencies), and make collaborative decisions (mechanism).	Contexts in which this is relevant include multidisciplinary teams who are not working on the same site; complex provider organizations with management teams and clinicians working on multiple sites; situations in which people may be receiving care from multiple teams, for example, from an inpatient or crisis service, as well as a continuing care service.	Making use of telemental health platforms to strengthen liaison and collaboration between teams and professionals on different sites (eg, through increased enhanced liaison between managers across an organization) or provide better access to a range of educational events Using telemental health platforms to facilitate multidisciplinary team meetings between staff on different sites (especially if some are working from home) However, awareness is needed that perceived pressure for staff to provide an immediate response may also negatively affect their work-life balance

^a^CMO: context-mechanism-outcome.

### Domain 3: Safety, Privacy, and Confidentiality

Table 3 presents the four overarching CMO configurations, key contexts, and example strategies and solutions for the domain of safety, privacy, and confidentiality. Key messages were the importance of ensuring the availability of a private space for both service users and clinicians (CMO 3.1), the potential for telemental health to provide privacy to some service users experiencing stigma (CMO 3.2), the importance of considering how to manage risk when using telemental health and the limits to how far this is possible (CMO 3.3), and data security and staff training (CMO 3.4).

**Table 3:** Domain 3: Safety, privacy, and confidentiality.

CMO^a^ title	References	Overarching CMO	Key contexts	Example strategies and solutions
CMO 3.1: Lack of privacy	[39, 42, 45, 48, 54, 55, 58, 60, 77, 79-82, 90, 91, 97, 101, 102, 104, 111, 120, 122-127]	When accessing telemental health sessions without access to a private space or secure private connection (context), service users and staff are at an increased risk of being overheard (mechanism 1), potentially leading to breaches of privacy and confidentiality (outcome 1), risk of harm to those in unsafe domestic situations (outcome 2), and reluctance to speak openly about sensitive topics (outcome 3). It may also cause some service users to experience frustration, distress, and anxiety (mechanism 2), leading to impacts on service user engagement and interactions (outcome 4) and reduced willingness to use telemental health and continue therapy (outcome 5).	Issues related to lack of privacy at home are especially relevant for young people who are distracted by their home environment, may not feel safe in their own home, or have siblings/parents/other family members unexpectedly appearing in the room; parents with children at home; those experiencing domestic abuse who are not able to be honest about symptoms, risk, or violence experienced; people who may be living with/caring for extended family or in households that are crowded; inpatients who may not have a space where they feel *psychologically safe*; people living in houses of multiple occupation; staff members who are not able to work in a private environment when providing remote therapy; and service users who experience cultural stigma in the home from their families relating to their mental health.	Brainstorming with the service user whether there are potential options for private places or times when privacy is more likely Offering face-to-face sessions when a private space is not available for telemental health, especially if there is any possibility that the person is at risk from someone in their home environment Regular, discreet checking that the service user (and therapist) is in a private space (eg, using the chat function in video calls) and taking steps to provide alternative locations if not Being flexible regarding the time of appointments Allowing people to turn off their camera or use virtual or blurred backgrounds (as well as ensuring that the option is available and they are aware of how to do this) Working with schools to provide safe spaces away from home for children (although young people may not want to alert teachers/other pupils to their need for a space to use for therapy) Attention to clinicians’ access to a private space and disclosure to service users if they are not in a completely private environment (eg, a shared office or a private home with other family members on the premises) Use of headsets with microphones
CMO 3.2: Privacy, anonymity, and reduced stigma (*service users*)	[40, 43, 92, 103, 105, 128-131]	For some service users who feel stigmatized when attending a mental health service in person and who have access to a private and secure space to receive therapy remotely (context), being provided with the option of telemental health as an alternative means there is an option to receive care with more anonymity (mechanism), which helps ensure their privacy and safety (outcome 1), thereby increasing the accessibility of services (outcome 2).	Some groups may be more likely to feel there is a stigma associated with attending mental health premises or reluctant to have contact with others doing so, for example, young people not previously in contact with services.	Offering telemental health (or home visits) to avoid missed appointments to people who are reluctant to attend mental health premises because of perceived stigma or as they find them intimidating
CMO 3.3: Managing risk	[24, 43, 85, 91, 111, 112, 119, 124, 132-136]	When services incorporate tailored risk management procedures in the delivery of remote care (context), it encourages consideration of the risks associated with remote care specific to each individual, including the risk of self-harm or suicide and risk from others in situations of domestic abuse, and ensures the staff are aware of the procedures to try to assess and respond to risk or safeguarding concerns despite challenges associated with remote care (mechanism), which has the potential to improve the safety and well-being of service users and others (outcome 1). However, a disadvantage of telemental health is that real-time risk assessment limits an immediate response to be organized when someone is at imminent risk of harm and some distance away (outcome 2).	This may be relevant to people who are currently unwell or in a crisis, situations where someone is remote from the assessing clinician or at a location unknown to them, situations where technological difficulties occur during an assessment of someone who is at high risk, people with eating disorders or who are physically unwell and where there are practical impediments to assessing risk remotely, and when a service user suddenly exits during a telemental health consultation and it is not clear why. In substance misuse services, it may be harder to detect whether someone is under the influence of drugs or alcohol.	Establishing a call back number before the commencement of the session in the case of disconnection when discussing distressing or sensitive topics Increased coordination with service users/families to facilitate safe transport to emergency departments if needed Setting clear protocols regarding when staff can be contacted via digital means, including who to contact instead in the case of an emergency Identification of where the service user is located at the start of the session to enable a faster response of in-person support if needed Development of a “telehealth manual” containing information on what to do in the event of a sudden ending of the call and who to contact Codevelopment of a crisis plan with the service user Offering 24/7 helplines and continued availability of face-to-face crisis response, including capacity for home visits Ensuring adequate device battery or connecting to a charger at the start of the session to reduce the risk of disconnection
CMO 3.4: Technological support and information security	[85]	When services provide technology support, software with appropriate security, and devices (including mobile phones and headphones) to staff specifically for work use (context), it helps ensure privacy and confidentiality for both service users and staff (outcome), as staff can store information securely on devices that are not shared with others (mechanism 1) and are able to ensure that service users are aware of when they will have access to their work devices (mechanism 2).	This may be relevant in services where staff share office space and devices, or where a shortage of devices may lead to the use of personal devices, for example, for home working; when balancing service user preference with risk from using less secure software or software with which the staff are less familiar; where software has a particular set of settings that must be enabled to ensure secure, private connections.	Providing data safeguarding and other technology-based training to all staff (as knowledge cannot be assumed) Providing information on which software is encrypted/secure Providing funding to staff for the purchase of equipment Setting recommended boundaries for both service users and clinicians in relation to the privacy of personal life and maintaining a work-life balance, for example, by not being contacted outside working hours or using a personal phone

^a^CMO: context-mechanism-outcome.

With the most supporting literature, CMO 3.1 highlights the need for appropriate private space to receive telemental health and that many service users may not have consistent access to such a space. As a lack of privacy can risk breaches in confidentiality and safety for some, a key message was that alternatives such as face-to-face modalities or alternative times/locations to receive telemental health should be provided. The importance of privacy for effective mental health care has been frequently cited in the literature and is likely to be especially important in ensuring high-quality telemental health [91]. Although some literature indicated that some service users feel an increased sense of privacy and a reduction in stigma when not having to attend mental health clinics in person (CMO 3.2), a key message lies in providing a choice so that each individual can work with their clinicians to find ways of receiving care that they are happy with, a message highlighted in CMO configurations throughout this paper.

CMO configurations in this domain also make it clear that telemental health can result in greater risks, both directly as it may be more difficult for clinicians to assess and respond to risks (CMO 3.3) and indirectly if data security is impaired (CMO 3.4). In both cases, proactive steps to assess and limit risk before the use of telemental health, as well as preplanned strategies to respond to events that threaten safety, are important. Data security knowledge should not be assumed, and training to help staff keep service users’ personal information secure will also mitigate telemental health–specific risks. However, evidence from both the literature and the stakeholder consultations made it clear that it is difficult to fully overcome the obstacles to effective risk assessment and management that result from staff and service users being in different places, meaning that the continuing availability of an in-person community crisis response is also important.

### Domain 4: Therapeutic Quality and Relationships

Table 4 displays the overarching CMO configurations, key contexts, strategies, and solutions for the final domain of therapeutic quality and relationships. Therapeutic relationships have been identified as pivotal for the successful delivery of telemental health across the literature and stakeholder consultations. The domain addresses barriers (CMO 4.1 and CMO 4.2) to and facilitators (CMO 4.3, CMO 4.4, CMO 4.5, and CMO 4.6) to the development of therapeutic relationships and delivery of quality care and discusses the impact of telemental health on staff well-being (CMO 4.7).

Trust and therapeutic relationships are important across health care, and relational aspects of care are especially crucial in mental health [173-178]. However, the reliance on telemental health platforms, particularly telephone and text-based communication, may affect communication and subsequently therapeutic relationships (CMO 4.1). Our CMO configurations, particularly their mechanisms, were informed by general theories regarding the role and development of therapeutic relationships in mental health care.

CMO 4.1. highlights that telemental health is likely to lead to a change or reduction in visual and nonverbal cues, including active listening and back channels, facial expressions, gestures, posture, and eye contact, which makes aspects of communication, such as pauses, difficult to interpret. In addition, time delays in video calls may create silences and lead to talking over each other and delayed visual responses, which negatively affect communication and nonverbal synchrony [179,180]. As a result, not only therapeutic relationships but also the staff’s ability to conduct accurate assessments are compromised (CMO 4.1 and CMO 4.2). However, conversely, good quality video contacts with well-trained clinicians may mitigate some of the therapeutic challenges when delivering telemental health. Those making first contact with mental health services appear to be particularly affected by the potentially impersonal nature of telemental health and thus benefit not only from an initial face-to-face session but also from more frequent subsequent telemental health sessions to establish stability and trust (CMO 4.1 and CMO 4.5). In addition, our findings indicate that staff confidence and ability to deliver good quality care and develop therapeutic relationships via telemental health can be fostered through training sessions provided by services (CMO 4.3).

The literature and stakeholder consultations identified no telemental health modality that is consistently superior for developing therapeutic relationships (CMO 4.4). Rather, whether telephone calls, video calls, or face-to-face meetings are most appropriate seems to depend on the purpose of the sessions and on an individual’s preferences based on their personal experiences and circumstances and whether they are new to the service, as well as the nature of their mental or physical health problems. Video calls seem to be preferred for more substantial and in-depth sessions than other telemental health modalities [24]. Providing service users with choice regarding the frequency, duration, and telemental health modality is crucial for therapeutic relationships and quality of care.

Despite its limitations, flexible use of different telemental health modalities can provide significant opportunities to foster therapeutic relationships and increase the quality of care, such as checking in, sending reminders via SMS text messages, and using features such as chat functions to increase engagement among service users (CMO 4.6).

Finally, taking breaks in between telemental health sessions and fostering positive telemental health working environments is key for staff well-being and the delivery of high-quality care (CMO 4.7).

**Table 4:** Domain 4: Therapeutic quality and relationship.

CMO^a^ title		References	Overarching CMO	Key contexts	Example strategies and solutions
CMO 4.1: Change in nonverbal cues and informal chat, affecting the therapeutic relationship		[12, 19, 24, 36, 37, 40, 45, 49, 54, 55, 60, 67, 76, 77, 79, 81, 90-92, 96, 103, 137 - 142]	When switching from face-to-face to telemental health care (context), staff and some service users perceived the relationship between staff and service users (and other service user group members) to be negatively affected or found it more difficult to develop a therapeutic relationship (outcome 1) and, thus, were less willing to take up or use telemental health (outcome 2), more likely to be dissatisfied (outcome 3), and viewed care as less effective compared with previously received face-to-face care (outcome 4). This was because they perceived telemental health to be impersonal and found it more difficult to discuss personal information because of a lack of nonverbal feedback, eye contact, and social cues, as well as informal chat before, after, and during sessions (mechanism).	This may be relevant during rapid switches to telemental health because of emergency situations such as the COVID-19 pandemic in which staff training and structured telemental health implementation are limited because of time constraints; for staff with limited training and experience generally and those with limited experience of using telemental health specifically, who may lack the confidence to navigate the change in visual cues, which, in turn, can affect the therapeutic relationship; for staff and service users who are new to a specific service, staff/service user, or to mental health care generally; for service users who are apprehensive of technology use or who are concerned about the violation of their privacy; and in telemental health group sessions in which the flow of conversation is affected or people find it less easy to establish relationships and be at ease with the whole group.	Offering new service users the option to receive their first appointment face-to-face when starting telemental health, depending on their preference Under pandemic conditions, exploring whether service users prefer telemental health sessions over face-to-face sessions, which require wearing masks Checking in with service users about their experiences and preferences regularly while trying to use a particular telemental health platform consistently Allocating additional time to address service user concerns about technology use and privacy Using high-quality equipment and ensuring good camera placement during video calls Making greater efforts to communicate clearly, enhance gestures, and provide verbal and nonverbal reinforcement, such as active listening and backchanneling (ie, nonverbal or verbal responses) Focusing on service user–centered communication, such as being reassuring and supportive Taking more time to informally chat and get to know new service users one-to-one when delivering the initial appointment via telemental health Providing training to staff to increase their comfort with technology and training to interpret social cues when using telemental health Providing reassurance to staff that service users often perceive the therapeutic relationship to be less affected by telemental health than staff believe Facilitating relationships between service user group members by keeping the video call open after the main session to allow follow-up conversations
CMO 4.2: Assessment via telemental health versus face-to-face (*staff*)		[40, 82, 92, 111, 121, 133, 137, 138, 143 - 145]	When using telemental health for assessments (context), staff report finding it more difficult to assess mental health problems, care needs, and risk, and make diagnoses (outcome), as they are less able to observe nonverbal and visual cues (depending on the telemental health modality used), and some service users may find it more difficult to have in-depth conversations about their problems and experiences (mechanism).	Cues can include extrapyramidal symptoms from antipsychotics, hygiene, gait, direct eye contact, mannerism, and linguistic nuances. Conducting assessments might be particularly difficult over the phone because of the lack of visual cues; with service users who experience domestic violence and abuse and thus cannot be honest about their well-being and current situation in the presence of their abuser; with young children; with service users who find it difficult to speak directly about their difficulties and experiences; and when the staff make incorrect assumptions about service users’ mental states based on behavioral indicators and without considering service user reports, especially of neurodivergent service users.	Offering service users the option to receive face-to-face care for first assessments and in crisis situations Taking both service user reports and nonverbal and visual cues into account for assessments Offering service users experiencing domestic violence and abuse the option to use text-based communication in addition to face-to-face care or other telemental health modalities to avoid being overheard Providing training to staff in conducting assessments using telemental health
CMO 4.3: *Staff* support and training		[49, 63, 102, 146]	When staff receive specific instructions and training, for example, on how to build rapport using telemental health and support from colleagues with prior telemental health experience (context), it facilitates quality of care (outcome 1), building therapeutic relationships (outcome 2), and increased engagement (outcome 3), as staff are able to ask questions and acquire new skills and knowledge about the interventions and thus build confidence in delivering telemental health (mechanism).	This is likely to be especially relevant to staff who have little or no previous experience of delivering telemental health.	Offering staff training on aspects of good care that go beyond technical skills and issues Staff training should ideally be co-designed and codelivered with service users
**CMO 4.4: *Service users* who find it easier to establish a therapeutic relationship**					
	On the web	[39, 60, 79, 96, 99, 119, 136, 137, 147, 148]	When delivering telemental health to some services users who feel uncomfortable in clinical settings and social situations (context), these service users find it easier to build a therapeutic relationship and are more willing to use telemental health (outcome), as they feel safer, are more relaxed and less anxious being in their own environment or outside of clinical settings and in-person social situations, or both, and, thus, feel more empowered and comfortable to open up and speak freely (mechanism).	This may be especially relevant for some children and young people, including those with special needs and neurodivergent children, who find clinical settings and having to travel upsetting, and some service users with social anxiety. However, it is important that using telemental health does not reinforce potentially detrimental safety behaviors that may maintain and potentially exacerbate their social anxiety.	Offering service users the option of receiving care by telemental health rather than face-to-face, especially if they neither wish to attend clinical settings nor to be visited by professionals at home
	Via video versus phone	[24, 36, 50, 52, 55, 78, 105]	When service users and staff who prefer video calls use them (instead of telephone calls or text-based chats) for telemental health (context), it can facilitate a stronger therapeutic relationship (outcome 1), satisfaction (outcome 2), and engagement (outcome 3), as it is easier to see visual and nonverbal cues, gauge the therapist’s reaction, and connect with the service user/staff member than with other telemental health modalities (mechanism).	This applies to service users across age groups and may be especially the case for new service users.	Encouraging clinicians to offer video calls rather than relying on phone calls and SMS text messaging and providing the relevant infrastructure and guidance to support this
	Via the phone versus face-to-face or video calls	[50, 65, 80, 111]	When services offer phone calls and SMS text messages instead of video calls (context), some service users are more satisfied with their care (outcome) as they do not have to sit still and see themselves on screen, are less conscious of their body language and facial gestures, are less distracted by the clinician’s nonverbal cues, are able to move around freely, and are thus less inhibited and able to open up more quickly (mechanism).	This might be especially relevant for service users who are neurodivergent, socially anxious, and self-conscious about their appearance.	Informing service users about the option to turn off their camera during video calls or using the phone if they are uncomfortable Offering a telephone call or text service instead, if the service user prefers this
CMO 4.5: More frequent telemental health sessions plus SMS text messages		[39, 40, 99, 104, 149]	When services adapt flexibly to service users’ preferences regarding the pattern and frequency of telemental health sessions, including offering more frequent, shorter rather than infrequent, long sessions, and additional asynchronous SMS text messages and calls to check in between sessions (context), it may lead to stronger therapeutic relationships (outcome 1), increased engagement (outcome 2), and improved quality of care (outcome 3), as service users receive regular and more frequent support depending on their preference (mechanism).	Lack of a need to travel means that more frequent shorter sessions may be particularly feasible with telemental health: they are potentially less tiring and thus might better maintain concentration and engagement, especially for children. Frequent sessions might help new service users to build trust and reduce anxiety around the treatment. Frequent sessions may also help support and monitor less stable service users, for example, following a crisis.	Considering offering shorter and more frequent sessions when telemental health is a primary modality for delivering care
CMO 4.6: Enhancing the quality of care through the use of telemental health enhancements		[24, 74, 101, 150-152]	When clinicians make appropriate and personalized use of enhancements and extensions of telemental health (such as using chat, voice activation to instruct phones, SMS text messaging and other text-based messaging, web-based appointment schedules, screen sharing, and apps accessed during sessions; context); it can lead to success engaging in telemental health (outcome 1) and broadening the range of strategies and interventions available during clinical meetings (outcome 2), as these features make engaging with services easier and provide a functional method useful for exchanging practical information, such as reminding service users about the date and purpose of an appointment, with less room for ambiguity and more creative methods of engagement (mechanism).	Additional telemental health features might be particularly helpful for young children (who overall find it difficult to engage on the web). Adolescents who experience social anxiety or are autistic may benefit from and prefer the chat function.	Using SMS text messaging (including apps) to maintain communication in a flexible way between appointments, especially for younger people for whom this may be a preferred method of communication Using screen sharing to facilitate psychoeducation or working together on assessment or therapeutic tools Using telemental health sessions to introduce apps and websites that support self-management or therapy or to collaboratively complete measures and questionnaires
CMO 4.7: *Staff* well-being and quality of care		[51-53, 80, 88, 91, 101, 107, 112, 153]	When the staff use the time saved on travel to take breaks in between telemental health sessions (context), it may increase staff well-being (outcome 1) and improve quality of care (outcome 2), as it provides the opportunity to reflect and recharge after telemental health sessions, which are often experienced as tiring and thus reduces fatigue, tension, and anxiety among staff (mechanism 1), and staff can use some of the time on clinical work, catch up on administrative tasks, or engage in professional developmental activities (mechanism 2).	This may be relevant for clinicians who can work wholly or partly at home and teams working across different sites or who visit service users at home or in other community settings.	Supporting clinicians in planning their time so that they can work from home and save travel time on some days Considering appointing some interested professionals to fully remote roles in which they can develop skills and make efficient use of time Ensuring that when time is saved because travel is not needed, clinicians still have suitable breaks between on-screen appointments and are able to dedicate some of the time saved to their own professional development Fostering a working culture in which staff are encouraged to take breaks between telemental health sessions to reflect and recharge

^a^CMO: context-mechanism-outcome.

## Discussion

### Principal Findings

Our RRR identified CMO configurations within four key domains, each with a range of practical implications regarding what works and for whom in telemental health: connecting effectively; flexibility and personalization; safety, privacy, and confidentiality; and therapeutic quality and relationship. Potentially, the most important finding of this realist review is the significance of personal choice and that one size does not fit all for telemental health. This includes choice of modality (eg, video, telephone, and text-based chat functions), platform, frequency or duration of sessions, and the option to revert to face-to-face sessions if preferred or required by the service user based on their current context or to vary modality from contact to contact. This review has highlighted that there are many contexts where face-to-face care is preferred or needed by service users, and this should be accessible and available to them and should be of equivalent timeliness to remote care (especially when delivered as part of routine care rather than as a response to a national emergency). However, the use of telemental health is a convenient and potentially advantageous option for some people in many contexts; thus, it is beneficial for mental health clinicians to have the skills and resources to offer telemental health as an option. When service users’ choices about what works for them are respected and decisions about care planning are made collaboratively, it is likely to be conducive to a stronger therapeutic relationship where the service user feels heard and respected [178].

Access to a device with a stable internet connection, as well as the confidence and ability to use a device to access telemental health, were identified as minimum requirements for both staff and service users to access telemental health, without which face-to-face appointments would be necessary. The devices and platforms used for delivering telemental health needed to be user-friendly [163,164], and preferably familiar, to easily facilitate sessions. Telemental health seemed to reduce some barriers to receiving mental health support experienced by some service users, such as those who were unable to travel or in inpatient wards, making it an acceptable alternative to face-to-face sessions for some people under these circumstances. It may also potentially allow service users greater access to out-of-area specialist services and support that is focused on specific groups (eg, cultural or lesbian, gay, bisexual, transgender, and queer groups). Issues of privacy, including data protection and confidentiality, or staff and service user access to a private space were emphasized throughout the literature and our consultations; this is likely to disproportionately affect disadvantaged groups of service users, such as those experiencing poverty, those in multi-occupancy households, children and young people, or people living with controlling or abusive partners or other family members. Already disadvantaged groups are similarly at particularly high risk of inequalities being exacerbated through digital exclusion. The “inverse digital care law,” stating that the use of digital technologies can make health inequalities worse [154], may well apply to the widespread implementation of telemental health [16]. Service planning and delivery must be based on a strong awareness of these risks and the need to overcome these barriers. There is also a duty to ensure that in-person care of equivalent quality remains readily available.

The impact on therapeutic relationships for both staff and service users has also been highlighted, with difficulties interpreting visual or nonverbal cues being cited as a barrier to establishing a good therapeutic relationship that enables service users to disclose sensitive information and staff are able to conduct valid clinical assessments. Adapting to service user preferences flexibly and giving weight to self-reports during assessments is likely to increase the quality of care and foster strong therapeutic relationships.

### Strengths and Limitations

The use of the RRR methodology to rapidly establish a set of theories about what works, for whom, and in which circumstances in telemental health has several strengths. The breadth of screened written evidence extends beyond published academic literature to nonacademic (including policy, third sector, and lived experience) sources. The targeted call for evidence sent directly to expert stakeholders from research, policy, and clinical settings (nationally and internationally), the voluntary sector, lived experience groups, minority groups, and representatives from health tech initiatives identified resources that would otherwise have been missed. Through these procedures, we rapidly identified literature from a wide range of key perspectives to contribute to the development of the CMO configurations.

The analysis process was rigorous and valued both published literature and stakeholder views, with the use of rapid realist methods allowing a range of stakeholder perspectives to be incorporated beyond what is normally possible in reviews. Our expert reference group (including clinical, academic, and lived experience experts) fed into the review process and theory development throughout, iteratively reviewing the plausibility, relevance, and usefulness of our individual and overarching CMO configurations. A wider group of expert stakeholders provided further input to identifying sources and reviewing overarching CMO configurations, especially regarding our priority groups: children and young people, users of inpatient and crisis care services, and digitally excluded populations. Continuous detailed feedback from the lived experience researchers and frontline clinicians helped to reduce bias toward academic perspectives; ensured the inclusion of a breadth of real-life experiences; and supported the iterative development of our methods, results, and interpretation of the findings.

A final key benefit of the RRR methodology is that we could rapidly investigate not only outcomes of telemental health use but also the mechanisms underlying what works and for whom, which most methodologies do not allow. We could also explore the contexts in which telemental health was implemented, as well as the telemental health resources that are available. This approach should be considered for future evaluation of telemental health.

Some limitations should be noted. The first relates to generalizations made in the process of developing overarching CMO configurations. These tended to combine underlying CMO configurations that related to a range of service user and clinician groups, service settings and types, and social and national contexts. We looked for important themes that appeared of general relevance and were validated through stakeholder consultation. However, it is likely that in some areas, we lost a more nuanced understanding of the relationship between particular CMO configurations and particular contexts. We also used a broad definition of telemental health to capture as much richness (and data) as possible from the available sources. However, we have merged heterogeneous forms of telemental health within most of our overarching CMO configurations, which may differ in their effectiveness and underlying mechanisms. Therefore, the conclusions are limited regarding mechanisms; outcomes for specific types of telemental health; and the impact on service users, staff, and carers. We also included literature that draws on the experiences of service users and clinicians both before and during the pandemic. However, technologies and approaches to implementation have changed substantially, with prepandemic evidence tending to focus on the planned and relatively small-scale implementation of tools specifically designed for mental health. Studies from the pandemic tend to relate to a range of phone and video call technologies implemented at scale with limited strategic planning. During the pandemic, staff and service users may also have been more willing to trial telemental health, given the extraordinary circumstances. The available technologies, and clinicians’ and service users’ skills in applying them, are also likely to have changed over time and are likely to continue to change.

The nature and strength of evidence drawn on for the review also need to be noted. Most sources were qualitative studies, service evaluations, or cross-sectional studies of associations; we found few relevant trials or longitudinal studies. We tried to maximize the value of this body of evidence by combining findings from multiple studies with expert stakeholder input to obtain theories with multiple sources of support about what works and for whom, illustrating them with example contexts and strategies. However, the lack of testing through traditionally robust methods in testing intervention strategies, such as trials and other longitudinal forms of evaluation, still needs to be noted, as discussed further in the *Implications for Research* section. In line with realist methodology [27,29], we did not appraise the sources we identified using traditional methods. However, sources were only included if they provided sufficient information on context, mechanism, and outcomes and contributed to the development of our overarching CMO configurations. Our extracted data were reviewed for their validity and coherence by our expert reference group.

Despite the inclusive search strategy and specific efforts to gain a wide range of perspectives, digitally excluded groups remain underrepresented in this study. This is partly because of the lack of literature focused on digital exclusion and the web-based methods used to conduct our review during the COVID-19 pandemic. Efforts were made to gain perspectives on digitally excluded groups by involving charities and staff and service user advocates working with people in such groups, including in projects aimed at addressing digital exclusion, in our stakeholder consultation. However, people experiencing severe digital exclusion did not participate in our web-based consultations, and the extent to which others can advocate for them is limited. Similarly, this study identified a lack of evidence in the literature about how to make telemental health engaging and effective for young children, nor were we able to find many people with relevant expertise to participate in our consultations. Data were also limited on group therapy and the role or experiences of families and other supporters of service users. Most available literature focused exclusively on staff perspectives of telemental health and crucially neglected to include the views or experiences of service users and their families or other supporters. Therefore, we were unable to incorporate these groups and their perspectives in our analysis and synthesis.

This study was initially planned and commissioned through discussions between policy makers in the Department of Health and Social Care and the MHPRU leads; lived experience researchers did not have the opportunity to contribute during the early stages of formulating research questions and identifying the methodology to be used.

### Implications

#### Implications for Clinical Practice

A range of implications for clinical practice and service planning can be drawn from our CMO configurations. The challenge for the future will be to find sustainable ways of implementing them in clinical practice and finding an appropriate balance between telemental health and traditional face-to-face care in future service delivery. In the context of a recent emergency (the COVID-19 pandemic), telemental health has been used with some degree of success to maintain care for at least some service users. Evidence and experiences from this widespread emergency implementation are helpful, both to inform future responses to such emergencies and allow a preliminary assessment of potential opportunities and pitfalls in implementing telemental health beyond an emergency context.

Some clear principles to guide practice emerge from our CMO configurations. Offering choice, planning care collaboratively, and listening to personal preferences regarding whether to use telemental health need to be embedded within services in which there is continuous use of telemental health as we move through and out of the COVID-19 pandemic. How choice is negotiated, enabled, and communicated is crucial. For choice to be real, options need to be clearly explained and discussed at every stage; face-to-face care of equal quality should be delivered as promptly as telemental health; and choice should be seen as dynamic, especially when a service user is in crisis. Preferences should be reconfirmed regularly and hybrid forms of care made available if appropriate. Choices may also be different after the COVID-19 pandemic, when the risk of infection traveling to and at appointments may no longer be a concern and consultations are no longer masked; mask-wearing at most face-to-face appointments during the pandemic may undermine some advantages of in-person care. Ideally, service user and clinician choices and resources should be balanced through shared decision-making. Use of telemental health cannot be assumed to be a permanent switch; thus, preferences should be revisited regularly. In planning services, it may be easier to switch from in-person appointments to digital appointments than vice versa, and this needs to be considered in staffing and working space arrangements. Traditional inpatient and community services are limited in their ability to collaborate and provide choice in their established processes, such as care planning and risk assessment or management [177,178,181-183]. Therefore, it may be unrealistic to expect improvements in these areas when delivering telemental health.

Lack of access to digital devices or data, or of expertise in connecting to telemental health services, is a problem that service providers may be able to address for some people. For example, opportunities to develop skills and clear guidance and opportunities to practice may be relatively straightforward ways of alleviating problems with connecting effectively for some people who may find telemental health a convenient way of receiving care if they are supported to engage. At best, obtaining access to telemental health may be a skill acquired along with developing the skills to access a variety of other significant parts of the digital world. In other instances, clinicians and service providers should be aware that digital exclusion tends to be rooted in other forms of disadvantage and that they can most readily avoid exacerbating such disadvantages by offering face-to-face care. Persevering with telemental health when service users do not want to receive care by this means and are not in the habit of using digital technologies may prove futile in everyday clinical care. There is also likely to be scope for improving the extent to which service providers have the capacity to connect effectively, for example, through better infrastructure, clear guidance and training for staff, and clarity and flexibility regarding platforms.

Developing a therapeutic relationship is key for the quality and success of care, and offering initial appointments face-to-face may facilitate this, subject to service user choice. In addition, services and staff may need to consider how to adapt telemental health care to account for the change in visual cues, including body language and facial expressions (although visual cues in face-to-face sessions may, in any case, be compromised while infection control considerations mean most sessions are masked).

In addition, the domain of privacy, safety, and confidentiality has implications for clinical practice. Clinician awareness of potential risks associated with using telemental health is important and may steer them away from conducting some consultations in this way. Maintaining privacy and safety, for example, for people at risk within their homes, is also a significant reason to prioritize service user choice, especially choices not to accept telemental health appointments, as they may not readily be able to explain the basis for their choice. Clinicians need also to be aware of the challenges of assessing and responding to risk when using telemental health, including the advantages of face-to-face meetings; the need to give weight to service user reports where visual or verbal cues may be obscured; and the importance of backup plans, such as for disconnection or when an urgent response is needed. Clinicians and care coordinators could also helpfully ensure that service users have access to and can use telemental health care adequately before the agreed web-based sessions begin, although this may be affected by staffing issues and limited resources [184,185].

#### Implications for Policy

Digital poverty does not exist in isolation, and the experience of poverty may be the root cause of their digital exclusion. Providing service users with access to devices and the internet, for example, serves as an adequate short-term fix but does not address the systemic welfare issues experienced by many service users [186]. Strategies to mitigate digital exclusion could include the provision of good national Wi-Fi coverage, free broadband [187], and investment in accessible and connected community hubs; implementing these would require action from the government rather than health services.

Telemental health services seem to be a viable alternative to face-to-face care for some service users, including in emergency situations, such as COVID-19. To provide good telemental health services, investment is needed, for example, in providing telemental health–specific training and guidance, high-quality infrastructure, and potentially technological devices to staff and service users. Preregistration education and training should include skills in telemental health. Further investment is likely to be needed in updating this as evidence and technologies change (eg, to cover the ongoing costs of keeping hardware and software up to date). This may need to be balanced against any savings anticipated from implementing telemental health. This study has also highlighted the importance of service user and frontline staff involvement in the planning of all telemental health services and provision.

#### Implications for Research

Much of the included research was based on explorations of views and experiences of people participating in telemental health in various settings. We found few studies involving systematic evaluation of planned strategies to achieve high-quality implementation of telemental health in routine settings. Primary studies of this form would be valuable, potentially using implementation research and participatory action research models to explore outcomes and experiences of strategies aimed at good quality implementation of telemental health in varying real-world settings during and after the pandemic. Our CMO configurations have the potential to inform such a primary research study: it would be helpful to develop and test coproduced strategies for implementing principles encapsulated in the CMO configurations in real-world settings. Similarly, our understanding of what works and for whom in telemental health would be improved by conducting primary research with specific groups, particularly those excluded from previous studies, such as digitally excluded groups or peer support networks, and in specific contexts, such as in group therapy sessions. Identifying methods of reaching digitally excluded populations in research studies, as well as identifying groups for whom telemental health is not appropriate, would be helpful. This is likely to be labor and time intensive and needs appropriate funding. Future research could also usefully explore the use of different telemental health modalities individually and in more depth.

Choice has been emphasized as crucial in the use of telemental health. The mechanisms behind choices and collaborative decision-making would benefit from further investigation, potentially using realist methods and drawing on principles from shared decision-making research. Investigation is warranted of the best approaches to providing the information needed to make an informed choice, holding collaborative discussions on how to personalize care for each individual, and providing staff and service users with guidance and training needed to participate effectively in telemental health. At a provider level, evidence is needed on what makes a good telemental health platform, how to balance data security with the flexibility that service users and clinicians may value in choosing platforms and using familiar tools if possible, and how to adapt risk management to a telemental health context. However, trusts may compromise their ability to offer choice and flexibility to service users when they specify the platforms that can and cannot be used to deliver telemental health services (although this may have advantages, including increasing staff familiarity). Future work could helpfully investigate whether certain combinations of features, platforms, or modalities are optimal for service users, their carers, and clinicians.

Researchers conducting evaluations of telemental health should consider that *satisfaction* tends to be evaluated as one component. However, satisfaction with telemental health comprises several components that need to be individually considered. For example, the skills of therapists may be rated highly, whereas telemental health platforms themselves may cause significant frustration and, if they were scored separately, would be poorly rated. Therefore, telemental health needs to be evaluated as several elements rather than as a singularity.

The impact of telemental health delivery on staff is a further key area of investigation. Some staff in research studies and in our stakeholder consultations reported finding prolonged screen use draining and perceived it as a contributor to burnout. The impact of telemental health on staff and ways of ensuring that it does not increase burnout or physical or psychological stress requires investigation. It may be pertinent to investigate whether services function better, and staff and service users are more satisfied, when certain staff become telemental health specialists, as opposed to asking all users to engage with it for some appointments.

A final key consideration is that any future research into telemental health should include lived experience knowledge, expertise, and views, including those from digitally excluded groups. Working remotely as a research team can facilitate the inclusion of a range of key stakeholders who might otherwise not be able to attend face-to-face meetings, promote ongoing communication, and simplify the coordination of tasks. However, similarly to telemental health care, it can also lead to the exclusion of already marginalized groups, such as digitally excluded people. There is a need to fund research designed and led by people with lived experience of mental health service use.

### Lived Experience Commentary

The lived experience commentary written by KM, RRO, and PS is shown in Textbox 1.

**Lived experience commentary.**

**Commentary**

We welcome the question “what works for whom, in what circumstances, and how?” At its heart, a realist review understands that each person has different needs from services, including telemental health services. The challenge is the reliance on existing knowledge and the potential to overlook gaps, especially where the world has changed rapidly because of the COVID-19 pandemic.

The digital methods used to consult a wider audience also further marginalize everyone who does not have, or want, such access. Including people who do not use telemental health would produce different research questions and answers. Similarly, including technology experts might provide some reassurance, for example, about regulation, risk, and ethics raised by practices such as the recent sharing of free text data from a UK crisis text line with third-party researchers to develop artificial intelligence–driven tools [188].

Digital technology has increased restrictive practice in mental health via surveillance [189], sometimes based on poor-quality research conducted with financial incentives from manufacturers [190]. Health data have been shared with police in programs such as Serenity Integrated Mentoring on shaky legal and ethical grounds [191]. Although these are not telemental health per se, they provide a context. In that context, we would have liked the question “What works for whom?” to consider political and financial interests.

This study’s methods encouraged a discussion of choice, personalization, and flexibility, which we welcome. We highlight two reflections.

First, choice is not only about preferring one option over another: it can be life or death. Within mental health, service users are often expected to bare our souls to get our choices respected. With telemental health, this is dangerous. If the criteria for accessing a face-to-face service are harm-based, we might be forced to put ourselves at risk to obtain what we need. When someone is being abused by their partner, they may need face-to-face services but not explain why at a first assessment. We must be taken at our word without being required to explain ourselves to clinicians who have not yet earned our trust.

Second, choice is limited by the available options, which are constrained by material circumstances and power. Service users generally have relatively little power in their relationships with an overstretched system. If a wheelchair user’s choice is to travel to a building with an unreliable lift versus telemental health, it is not a meaningful choice. If you have to wait 6 months for a face-to-face appointment but you can have telemental health the next week, it is not a meaningful choice. If you cannot afford to connect to the internet, you do not have a meaningful choice. The option of telemental health must not become an excuse to allow face-to-face services to become harder to access.

Many of the actions for telemental health implementation are specific applications of general principles of good care: informed consent to make meaningful choices; clarity about the use of our health data, breeding trust; and understanding and responding to the contexts in which we live our lives.

Within such contexts, we welcome a focus on digital poverty as poverty. The policy solutions to poverty lie well beyond mental health—a broader overhaul of the punitive welfare system and a society in which workers are empowered to negotiate livable wages.

## Appendix

Multimedia Appendix 1 Glossary.

Multimedia Appendix 2 Study characteristics.

Multimedia Appendix 3 Overarching context-mechanism-outcome configurations.

## Abbreviations

CMO: context-mechanism-outcome
LEWG: Lived Experience Working Group
MHPRU: Mental Health Policy Research Unit
NHS: National Health Service
NIHR: National Institute for Health Research
PRISMA: Preferred Reporting Items for Systematic Reviews and Meta-Analyses
PROSPERO: International Prospective Register of Systematic Reviews
RRR: rapid realist review
