# Supplementary material for: Synthesis of the Evidence on What Works for Whom in Telemental Health: Rapid Realist Review
Source: Interact J Med Res. 2022 Sep 29;11(2):e38239. doi: 10.2196/38239 (PMC9524537; doi:10.2196/38239)
Supplement: Multimedia Appendix 1 [file ijmr_v11i2e38239_app1.docx]

# Appendix 1. Glossary

**Realist review (or realist synthesis)**

A theory-driven evidence review that aims to understand and unpack the underlying causal mechanisms by which a complex intervention (or ‘programme’) works, or fails to work. It provides an explanation of how and why the intervention works, for whom, and in what circumstances or contexts.

**Rapid realist review**

A type of realist review that focuses on producing practical, actionable findings that are useful to policymakers in responding to time-sensitive and/or emerging issues in cases where time and resources are limited. Consultation with stakeholders and experts is used to identify the focus of the review, define the research questions, and streamline the review process.

**Context**

Describes those features of the situations into which a programme (e.g. an intervention or policy) is introduced that affect how and whether the programme works. Context is broader than a specific healthcare setting or clinical population and may include, for example, programme resources and infrastructure, organisational structures, rules and processes, features of the social, political and economic environment, and cultural values and norms.

**Mechanism**

An underlying causal process or structure that gives rise to intended and unintended outcomes of a programme, within a particular context. Mechanisms are distinct from a programme’s components, inputs or outcomes. They describe how and why programmes contribute to outcomes and may operate at different levels of a system. They are often multiple and, in the case of, for example, psychological and social mechanisms, not directly observable.

**Outcome**

The outcomes of a programme can be intended or unintended, and short-, medium- or long-term. A programme may produce multiple outcomes of different types and of varying importance for different stakeholders.

**Programme theory**

A theoretical framework that explains how the activities and resources of a programme lead to particular outcomes in particular contexts. An intervention's programme theory is iteratively tested and refined during the course of conducting a realist review, drawing on evidence and theory from a range of sources and disciplines, such as psychology, sociology, education, economics and organisational behaviour. The refined programme theory becomes the final product of the realist review.

**CMO configuration**

CMO (Context-Mechanism-Outcome) configurations constitute the programme theory of a realist review (see above). An individual CMO configuration is a statement, sometimes represented by a diagram, that states the hypothesised relationship between a particular context, a particular mechanism and a particular outcome of a programme. It should be understood as meaning: “In this context (C), this mechanism (M) generates this outcome (O).” A CMO configuration may relate either to the programme as a whole, or only to a certain aspect of it. CMOs may be configured in a series, in which the outcome of one CMO becomes the context for the next. Within a realist review, they are used in different ways, for example, to frame review questions, to predict outcomes in different contexts, and as a framework for analysis.

**Underlying and overarching CMO**

In the current review, underlying CMOs are the CMO configurations that have been extracted from the individual studies included in the review. This resulted in a large number of underlying CMOs, which were then iteratively combined (to eliminate duplication), synthesised and refined to develop a smaller number of overarching CMOs relating to themes or domains judged to be of particular relevance for the review. Drawing on evidence and theory from the wider literature, and in consultation with a range of stakeholders, the overarching CMOs were then further refined and constitute the review’s final programme theory.
