# Supplementary material for: Synthesis of the Evidence on What Works for Whom in Telemental Health: Rapid Realist Review
Source: Interact J Med Res. 2022 Sep 29;11(2):e38239. doi: 10.2196/38239 (PMC9524537; doi:10.2196/38239)
Supplement: Multimedia Appendix 2 [file ijmr_v11i2e38239_app2.docx]

# Appendix 2. Study characteristics

| **Author, year and country** | **Study aim** | **Type of report and study design** | **Type of service** | **Nature of mental health problem/diagnosis** | **Participants (n)** | **Telemental health modalities** |
| --- | --- | --- | --- | --- | --- | --- |
| Aafjes-van Doorn et al. (2020) USA | Exploring therapists’ experiences of switching to video therapy during the pandemic. | Quantitative cross-sectional study | Psychology/psychotherapy/counselling service | Not applicable (NA) | Counsellors and Psychologists (n=141) | Video call |
| Adamou et al. (2021) UK | Investigating if tele-communication methods are preferable to in-person consultations at an Autism and ADHD service during the pandemic. | Quantitative cross-sectional and qualitative survey | Community mental health teams and outpatient services | ADHD and/or autism | Service users (n=117) | Video call, Phone call, Other (combination) |
| Arighi et al. (2021) Italy | Describing the digital divide of patients with dementia contacted by telemedicine during the COVID-19 pandemic. | Quantitative cross-sectional study | General hospital/physical health service | Dementia | Service users (n=108) | Video call |
| Bambling et al. (2008) Australia | Exploring the experience of online counsellors, particularly of text-based communication. | Qualitative focus group study | Helplines | General population | Counsellor (n=26) | Video call, Text message/SMS/WhatsApp |
| Barney et al. (2020) USA | Reporting on a clinic’s rapid transition to audiovisual telemedicine in response to COVID-19. | Service evaluation/audit: Quantitative cross-sectional study | Community mental health teams and outpatient services | Medical conditions, attention and mood disorders, sexual and reproductive health care, eating disorder care, addiction treatment | Mixed groups of mental health professionals  (n not stated/unclear) | Video call |
| Benaque et al. (2020) Spain | Describing the experiences of a service in dealing with the COVID-19 pandemic. | Service evaluation/audit | Voluntary sector/non-profit organisation | NA | NA | Video call, Phone call, Text message/SMS/WhatsApp |
| Bierbooms et al. (2020) The Netherlands | Discussing the expectations regarding the sustainability of online treatment post-COVID-19 regulations. | Qualitative interview study | Community mental health teams and outpatient services | Light to complex psychological and psychiatric problems | Mental healthcare staff (n=9), support staff (n=2) | Video call, online modules, Text message/SMS/WhatsApp |
| Boldrini et al. (2020) Italy | Investigating factors influencing the rate of interrupted treatments during lockdown and psychotherapists’ satisfaction with telepsychotherapy. | Quantitative cross-sectional study and qualitative survey | Community mental health teams and outpatient services; Psychology/psychotherapy/counselling service; Private hospital/clinic | Mixed primary care or psychology/psychotherapy service users | Psychotherapist or analyst (n=308) | Video call, Phone call, Mixed F2F and telemental health |
| Bommersbach et al. (2021) USA | Identifying staff perceptions on changes that should be maintained or improved in services. | Quantitative cross-sectional study and qualitative focus group study | General hospital/physical health service | NA | Staff (n=99 in survey; n=25 in focus group) | Video call, online patient portals |
| Brooks et al. (2013) USA | Describing the cultural adaption model. | Service evaluation/audit | Community mental health teams and outpatient services | PTSD | Service users (n not stated) | Video call |
| Buckman et al. (2021) UK | Exploring 1) changes of psychological therapy during the pandemic; 2) clinicians’ experience and confidence of delivering video-based psychological therapy; and 3) barriers, benefits, and training needs in respect to video-based psychological therapy delivery. | Quantitative cross-sectional study | Community mental health teams and outpatient services | NA | Therapists (n=66) | Video call |
| Castillo et al. (2020) USA | Describing the development of TeleMOUD tool (medications for Opiod Use disorder) to mitigate disruptions in care for people with Opiod Use Disorder. | Quantitative cross-sectional study;  descriptive study; Implementation study | Community mental health teams and outpatient services | Opioid dependency and drug injection | Service users and individuals who inject drugs (n=22) | Video call, Phone call |
| Chen et al. (2020) USA | Reviewing payment and regulatory changes in telepsychiatry in March and April 2020. | Commentary/editorial (with data) | General hospital/physical health service. | Mixed secondary mental health service users | NA (description of service change) | Video call, Phone call |
| Choi et al. (2014) USA | Evaluating the acceptance and preliminary efficacy of in-home telehealth delivery of problem-solving therapy (tele-PST) among low-income homebound older adults | Quantitative randomised controlled trial (RCT) | Aging service agencies | Depression | Service users (n=121) | Video call, Phone call |
| Chong and Moreno (2012) USA | Comparing telepsychiatry services through the Internet in primary care settings. | Quantitative randomised controlled trial (RCT) | Community mental health teams and outpatient services | Depression (n=167) | Service users (n=167) | Video call |
| Colle et al. (2020) France | Assessing the short-term acceptability of teleconsultation during the COVID-19 pandemic. | Letter: Quantitative cross-sectional study | Community mental health teams and outpatient services | Psychosis and bipolar, depression, anxiety, obsessive-compulsive disorder, stress, and substance misuse | Psychiatrists (n=17) | Video call, Phone call |
| Conn et al. (2013) Canada | Evaluating a telepsychiatry programme for older adults. | Service evaluation/audit: Quantitative retrospective and cross-sectional cohort study and qualitative focus group study | Community mental health teams and outpatient services | Mixed secondary mental health service users | Physicians (n=29, in survey), mixed groups of mental health professionals (n in focus groups unclear), service users (n=100 in retrospective chart review; n=253 sessional evaluation forms of patient assessments; n=41 case consultations) | Video call |
| Connolly et al. (2021) USA | Describing Veterans Affairs’ rapid increase in telemental health due to COVID-19. | Service evaluation/audit | Community mental health teams and outpatient services | NA | NA (description of service change) | Video call, Phone call |
| Costa et al. (2021) USA | Assessing resilience and mental health care during the COVID-19 pandemic, with a focus on telemental health. | Quantitative cross-sectional study and qualitative survey | Service user-led/peer support | Mixed primary care, psychology/psychotherapy, or secondary care service users | Service users (n=435 of which n=381 had self-reported mental illness) | Video call, Phone call |
| Crowe et al. (2016) USA | Comparing in person and telepsychiatry for deaf or hard of hearing patients. | Quantitative non-randomised controlled trial (nRCT) | Community mental health teams and outpatient services | Mixed secondary mental health service users | Service users (n=24) | Video call |
| Disney et al. (2021) USA | Evaluating the transition of a refugee-serving mental health clinic to telemental health as well as perceived obstacles and helpful resources. | Qualitative survey | Refugee-serving outpatient mental health clinic in a large refugee resettlement area | NA | Mixed groups of mental health professionals (n=17) | Phone call, or phone call in combination with video call (did not use video call alone) |
| Dores et al. (2020) Portugal | Analysing 1) changes in professionals’ use of and attitudes towards Information and Communication Technologies during the first COVID-19 lockdown; 2) factors underlying such changes; and 3) possible adoption of guidelines. | Quantitative cross-sectional study | Psychology/psychotherapy/counselling service | Depression, anxiety, neurodevelopmental, “personality disorders”, substance use disorders, sleep disorder, neurocognitive disorders | Psychologists (n=108) | Video call, Phone call, Email, Other (audio conferences, online intervention platforms, smartphones and tablet apps, online forums, chats, short-message services, virtual rooms), Text message/SMS/WhatsApp |
| Eagle (2020) UK | Exploring what has been advantageous and disadvantageous about remote therapy. | Service evaluation/audit and Primary research study: Qualitative interview study | Community mental health teams and outpatient services | Mixed secondary mental health service users | Service users (n=8) | Phone call, video call |
| Fogler et al. (2020) USA | Describing how children’s hospitals used treatment fidelity to guide adaptations to an ADHD bootcamp for parents. | Service evaluation/audit: Quantitative cross-sectional study and qualitative focus group study and survey | Tertiary care children’s hospitals | ADHD | Caregivers of 20 children with ADHD | Video call |
| Foye et al. (2020) UK | Exploring how Covid-19 has affected the mental health nurse workforce. | Quantitative cross-sectional study and qualitative survey | Community mental health teams and outpatient services; Crisis and emergency mental health services; adult and older adult services; children and young people; intellectual disabilities; forensic services; perinatal; drugs and alcohol services eating disorders | NA | Mental health nurses (n=897) | Video call, Phone call |
| Frayn et al. (2021) USA | Examining the impact of COVID-19 on people with binge eating spectrum disorders and their perception of tele-therapy. | Qualitative interview study | Community mental health teams and outpatient services | Eating disorders | Service users (n=11) | Video call |
| Gaddy et al. (2020) USA | Assessing the impact of the pandemic on the employment and service delivery of music therapy professionals. | Quantitative cross-sectional study and qualitative survey | Music therapy provided at schools, hospice, inpatient/outpatient psychiatry and private practice/contractual | Autism spectrum disorder, developmental disabilities, Alzheimer's disorder | Music therapists (n=1,196) | Video call, Phone call, Other (pre-recorded songs/playlists and video sessions) |
| Ghaneirad et al. (2021) Germany | Examining therapists' and service users' experiences of video consultations during the first COVID-19 lockdown. | Quantitative cross-sectional study | Community mental health teams and outpatient services | Mixed primary care or psychology/psychotherapy service users | Service users (n=338) | Video call |
| Ghosh et al. (2021) India | Describing the stepwise telemedicine model adopted by a Drug De-addiction and Treatment Centre. | Service evaluation/audit | Substance use clinic | Substance use disorder | Service users (n=379) | Video call, Phone call, Mixed F2F and telemental health |
| Godleski et al. (2012) USA | Assessing the feasibility and outcomes of the home electronic messaging programme for psychiatric patients. | Quantitative before-and-after study | Veterans’ healthcare community service | Schizophrenia, PTSD, depression and substance-use disorder | Service users (n=76) | Other (Electronic messaging device) |
| Greenwood et al. (2004) Australia | Evaluating a telepsychiatry clinical service in rural New South Wales. | Service evaluation/audit: Quantitative cross-sectional study | Satellite clinic in a rural site to bring specialized mood disorder | Mood and anxiety disorder | Service users (n=20) | Video call, Phone call, Mixed F2F and telemental health |
| Grover et al. (2020a) India | Evaluating the impact of the COVID-19 pandemic on mental health services. | Quantitative cross-sectional study | Community mental health teams and outpatient services; Inpatient mental health service; state-funded government medical colleges; private medical colleges; central government-funded institutes | Mixed primary or secondary care, or psychology/psychotherapy service users | Professors or heads of department from various medical colleges (n=109) | Video call, Phone call |
| Grover et al. (2020b) India | Evaluating the impact of the Covid-19 pandemic and lockdown on the mental health services in the private sector. | Quantitative cross-sectional study | Mental health services in the private sector (single chamber outpatient practice; mental health inpatient setting owned by professionals; mental health service provided through the corporate hospitals; multi-specialty hospitals) | General population in quarantine; mixed primary or secondary care, or psychology/psychotherapy service users | Members of the Indian Psychiatric Society working in the private mental health sector (n=396) | Video call, Phone call |
| Guinart et al. (2020) USA | Examining patients’ experiences of, and attitudes toward, telepsychiatry. | Quantitative cross-sectional study | Community mental health teams and outpatient services; General hospital/physical health service | NA | Service users (n=3,070) | Video call, Phone call, other (combination) |
| Hawke et al. (2021) Canada | Examining youth’s attitudes toward and experiences of virtual mental health services and substance use services. | Quantitative cross-sectional study and qualitative survey | Community mental health teams and outpatient services (CAMH specialty clinics) and no specific setting/general population | Mixed primary or secondary care, or psychology/psychotherapy service users | Service users (n=164), general population (n=245) | Video call, Phone call, Email, Text message/SMS/WhatsApp |
| He et al. (2020) China | Evaluating the 'The COVID-19 Psychological Resilience Model' intervention. | Quantitative cross-sectional study | No specific setting/general population | NA | NA (intervention description) | Video call*,* Phone call, Mixed F2F and telemental health |
| Healthwatch (2021) UK | Exploring the barriers to accessing mental health appointments by phone or video call. | Quantitative cross-sectional study and qualitative focus group study and Interview study | No specific setting | Mixed primary care or psychology/psychotherapy service users | Service users (n=98) | Video call, Phone call |
| Hensel et al. (2021) Canada | Exploring “how  videoconferencing should be used and for whom” with reference to postpartum women. | Qualitative interview study | Psychology/psychotherapy/counselling service within perinatal mental health service | Postpartum depression and anxiety | Therapists (n=3), service users (n=12) | Video call, Mixed F2F and telemental health |
| Hernandez-Tejada et al. (2014) USA | Investigating parameters and barriers to treatment completion among veterans receiving exposure therapies in-person compared to telemedicine*.* | Quantitative case–control study using secondary data | Not stated/unclear | PTSD | Veterans (n=47) | Phone call |
| Holland et al. (2020) UK | Providing guidance for remote working in mental health settings | NHS guidance document | No specific setting | NA | Not applicable (guidance document) | Video call, Phone call, Mixed F2F and telemental health |
| Hopkins and Pedwell (2021) Australia | Assessing the nature and impact of changes in child and youth mental health services. | Quantitative cross-sectional study and qualitative survey | Child and youth psychology/psychotherapy/counselling services | NA | Mixed groups of mental health professionals (n=113) | Video call, Phone call |
| Humer et al. (2020) Austria | Evaluating how well psychotherapists feel informed about the use of the Internet in psychotherapy and which software was used during the COVID-19 lockdown for online psychotherapy. | Quantitative cross-sectional study | Not stated/unclear | NA | Psychotherapists (n=1,547) | Video call |
| Johnson et al. (2021) UK | Describing the perspectives and experiences of staff working in inpatient and community settings. | Quantitative cross-sectional study and qualitative survey | NHS, private healthcare, social care, and voluntary sector services | Mixed primary care or psychology/psychotherapy service users, mixed secondary mental health service users, general population | Staff (n=2,180) | Video call, Phone call, Mixed F2F and telemental health |
| Jones et al. (2014) USA | Evaluating a technology enhanced version of behavioural parent training for children with disruptive behaviour from low-income families. | Quantitative randomised controlled trial (Pilot RCT) | No specific setting | Childhood disruptive behaviour | Families with low income (n=19) | Video call, Text message/SMS/WhatsApp |
| Juarez-Reyes et al. (2021) USA | Describing procedures, acceptability, and feasibility of converting from in-person to videoconference sessions. | Quantitative before-and-after study and qualitative focus group | University-based primary care clinic | Depression, anxiety, and/or stress | Service users (n=6) | Mixed F2F and telemental health |
| Kanellopoulos et al. (2021) USA | Describing perceived benefits and challenges of an inpatient Telemental Health conversion. | Letter with data on a psychiatric inpatient service | Inpatient mental health service | NA | Inpatients (n=15) | Video call, Phone call |
| Khanna et al. (2020) Australia | Investigating flexible work arrangements. | Service evaluation/audit | Community mental health teams and outpatient services | PTSD | Psychiatrists (n=4), registrars (n=3), psychologists (n=14) | Video call, Phone call, Mixed F2F and telemental health |
| King et al. (2006) Australia | Examining adolescents’ motivations and experiences in accessing the Internet for counselling services. | Qualitative focus group study | No specific setting/general population | Mixed primary care or psychology/psychotherapy service users | Service users and general population (n=39) | Text message/SMS/WhatsApp |
| Lakeman and Crighton (2021) Australia | Exploring the impacts of the cessation of DBT programmes and obstacles and solutions to providing DBT using telehealth technology. | Quantitative cross-sectional study and qualitative survey | 12 hospitals and 21 community health centres | “Borderline personality disorder” diagnosis | Mixed group of mental health staff (n=28) | Video call, Phone call, Mixed F2F and telemental health |
| Lecomte et al. (2020) Canada | Determining the feasibility and acceptability of CBT for first episode psychosis via videoconferencing. | Quantitative before-and-after study and qualitative interview study | Early intervention for psychosis service | Schizophrenia-spectrum disorders, bipolar disorder | Service users (n=14) | Video call |
| Liberati et al. (2021) UK | Exploring the experiences of service users, carers and staff seeking or providing secondary mental health services during the pandemic. | Qualitative interview study | Community mental health teams and outpatient services (early intervention for psychosis); Inpatient mental health service; Crisis and emergency mental health services; secure forensic services; specialist services | Mixed secondary mental health service users | Mixed mental health staff (n=35), service users (n=20), carers of people with mental health difficulties (n=10) | Video call, Phone call, Email, Text message/SMS/WhatsApp, Mixed F2F and telemental health |
| Lichstein et al. (2013) USA | Exploring the efficacy of telehealth targeting insomnia and depression in older adults in rural primary care settings. | Quantitative before-and-after study | Primary care CBT counselling service | Depression, comorbid insomnia | Service users (n=5) | Video call, Phone call |
| Lin et al. (2020) China | Evaluating the quality of a psychological hotline services. | Quantitative cross-sectional study | Helplines | NA | Staff (n=414) | Phone call, Text message/SMS/WhatsApp |
| Lindsay et al. (2015) USA | Investigating the implementation of video telehealth for the delivery of psychotherapy for veterans with PTSD. | Quantitative before-and-after study | Department of Veteran Affairs Medical Clinics | NA | 5 Veteran Affairs Medical Clinics | Video call |
| Lodder et al. (2020) UK | Evaluating procedural and implementation challenges of a blended, group-based psychosocial stigma protection intervention for parents of autistic children. | Qualitative focus group study and survey | Not stated/unclear | Autism | Children (n=9) and their parents (n=10) | Video call |
| Mad Covid (2020) UK | Discussing the impact COVID-19 has had on people with pre-existing mental health conditions and how mental health services can support them. | Service user led report: Qualitative focus group study and participant observation | No specific setting | Mixed primary care or psychology/psychotherapy service users and mixed secondary mental health service users | Service users, survivors, allies (4 panel members and 67 symposium attendees) | Video call, phone call |
| Mahmoud et al. (2021) USA | Examining approaches implemented by a telepsychiatry organisation to increase satisfaction, engagement and wellbeing among telepsychiatrists. | Quantitative cross-sectional study | Community mental health teams and outpatient services | NA | Telepsychiatrists (n=25 in 2018, n=23 in 2019) | Video call, Phone call, Email |
| Martin et al. (2020) UK | Investigating the role of digital communications in mental health services. | Qualitative interview study (secondary analysis) | Early intervention psychosis clinics | Psychosis and bipolar | Mixed mental health staff (n=19), youth service users (n=5) | Email, Text message/SMS/WhatsApp |
| McBeath et al. (2020) UK | Exploring the experiences and challenges of psychotherapists working remotely during the COVID-19 pandemic. | Quantitative cross-sectional study and qualitative survey | Not stated/unclear | NA | Psychotherapists (n=335) | Video call, Phone call, Email, Other (letters), Text message/SMS/WhatsApp |
| Medalia et al. (2020) USA | Describing key factors addressed by a clinic for adults with service mental illness to continue to provide services via video-supported telehealth. | Commentary/editorial with data | Community mental health teams and outpatient services | Severe mental illness | NA (description of service change) | Video call |
| Mental Health Commission of Canada (2014) Canada | Providing an overview of e-Mental health in Canada, including its challenges and barriers. | Briefing document for e-mental health care in Canada | No specific setting | NA | NA (briefing document) | Video call, Phone call, Email, Text message/SMS/WhatsApp, Other |
| Mental Health Network NHS Confederation (2020a) UK | Describing the experiences of two Mental Health Trusts delivering group sessions. | Briefing paper based on the experiences of two Mental Health Trusts delivering Group sessions | Community mental health teams and outpatient service; Psychology/psychotherapy/counselling service | Mixed secondary mental health service users | NA (briefing paper) | Video call, Phone call |
| Mental Health Network NHS Confederation (2020b) UK | Providing guidance for increasing service user choice in and access to digital mental health care. | NHS guidance document | No specific setting | NA | Not applicable (guidance document) | Video call, Phone call |
| Mind (2021a) UK | Reporting people’s experiences of being offered and using support from the NHS for their mental health by phone or online. | Quantitative cross-sectional survey and qualitative focus group and interview study | Mental health services (No specific setting) | NA | Mixed groups of mental health professionals (n=4 interviews), individuals offered NHS mental health support (n=1,914 survey participants, n=11 interviews); Mind’s ‘community activist’ campaigners (focus group, n not stated) | Video call, Phone call, Text message/SMS/WhatsApp |
| Mind (2021b) UK | Reporting people’s experiences of accessing support from the NHS for their mental health by phone or online. | Quantitative cross-sectional survey and qualitative interviews | Mental health services (No specific setting) | NA | General population accessing NHS mental health services (n=approximately 1,900 survey participants; n=5 interviews) | Phone call, video call |
| Moore (2021) UK | Presenting the challenges of people severely affected by mental illness have faced during the COVID-19 pandemic. | Briefing report: Quantitative cross-sectional study | Early intervention in psychosis & Liaison and diversion services | Severe mental illness | People with mental illness (n=272) | Video call, Phone call, Text message/SMS/WhatsApp |
| Moslehi et al. (2021) UK | Describing the Attend Anywhere platform, used in an inpatient psychiatric unit in Camden & Islington during the pandemic. | Service description | Inpatient mental health service | Mixed secondary mental health service users | NA (description of service change) | Video call |
| Newbronner et al. (2021) UK | Exploring the satisfaction with and difficulties accessing the support provided during the pandemic among people with severe mental illnesses | Quantitative cross-sectional study and qualitative survey | Primary care mental health trusts | Schizophrenia or delusional/psychotic illness or bipolar disorder | Service users (n=367) | Phone call, Text message/SMS/WhatsApp |
| National Health Service England (accessed 2022) UK | Describing the adoption of crisis lines in NHS services. | NHSE case studies of 24/7 crisis lines provided during COVID | 24/7 crisis helplines | NA | Not applicable (description of service change) | Phone call |
| Nissling et al. (2020) Sweden | Assessing the experiences of patients with anxiety disorders as well as the feasibility, safety, accessibility and effectiveness of an internet-CBT program. | Quantitative before-and-after study and qualitative interview study | Primary care | Anxiety disorder | Service users (n=9 for quantitative measures, n=8 for qualitative follow-up) | Phone call, Text message/SMS/WhatsApp |
| O'Dell et al. (2021) USA | Reporting the acceptability and usability of an InTouch platform used for Tele Behavioural Health | Service evaluation/audit: Quantitative cohort study (retrospective and cross-sectional) | Primary care | NA | NA (description of service change) | Text message/SMS/WhatsApp, Phone call |
| Ogueji et al. (2021) Nigeria | Exploring the willingness to utilise e-therapy services and identify its limitations in Nigeria. | Qualitative survey | No specific setting | NA | General population (n=100) | Video call, Phone call, Text message/SMS/WhatsApp |
| Olwill et al. (2020) Ireland | Reporting Irish psychiatrists’ experience of remote consultations at a Department of Old Age Psychiatry. | Quantitative cross-sectional study and qualitative survey | Outpatient psychiatric service, Department of Old Age Psychiatry | Mixed secondary mental health service users | Non-consultant hospital doctor and psychiatric consultants (n=26) | Phone call |
| Open Excellence (2020) USA | Publicising online hearing voices groups. | Publicity / news article | Service user-led/peer support: Hearing voices groups | Psychosis and bipolar | Peer workers (n=2) | Video call |
| Orlowski et al. (2016) Australia | Reporting the receptiveness and readiness of the youth mental health workforce towards the use of mobile and online-based technologies for young people. | Qualitative focus group study and Interview study | Youth Mental Health services | NA | Mixed mental health staff (n=40 in focus groups, n=8 interviewed) | Email, Facebook chat/messaging, Text message/SMS/WhatsApp |
| Peralta and Taveras (2020) Dominican Republic | Determining the effectiveness of teleconsultation during the pandemic in the Dominican Republic. | Quantitative cross-sectional study | Helplines | General population | NA (6,800 interventions) | Video call, Phone call, Text message/SMS/WhatsApp |
| Pierce et al. (2021) USA | Reporting the effect of the COVID-19 pandemic on the extend of telepsychology use. | Quantitative cross-sectional study | Community mental health teams and outpatient services; residential services; general hospital/physical health service; Psychology/psychotherapy/counselling service | Mixed primary care or psychology/psychotherapy service users | Licensed psychologists (n=2,619) | Video call, Phone call |
| Pote et al. (2021) UK | Reporting digital mental health competencies and training needed by UK clinical psychologists including opportunities and barriers. | Quantitative cross-sectional study and qualitative interview study | Psychology/psychotherapy/counselling service | NA | UK-wide Doctoral programmes in Clinical Psychology (n=18) | Video call, Email, Text message/SMS/WhatsApp |
| Pugh et al. (2020) UK | Investigation how chairwork is best provided in internet-delivered psychotherapy. | Qualitative survey | Not stated/unclear | NA | Mixed groups of mental health professionals (n=41) | Video call, Phone call |
| Rabinowitz et al. (2010) USA | Describing the development, implementation, and benefits of a nursing home Telepsychiatry consultation service. | Quantitative cohort study (retrospective) | Nursing home | Depression, dementia, other mental health disorders | Service users (n=106) | Video call, Phone call |
| Rosen et al. (2021) USA | Describing barriers, facilitators, and challenges of Veteran’s Health Administratoin expansion of telemental health care during the COVID-19 pandemic. | Service evaluation/audit | Veteran’s Health Administration mental health services in USA | Mixed mental health problems | NA (description of service change) | Video call, Phone call |
| Sasangohar et al. (2020) USA | Reporting the rapid implementation of telepsychiatry in a psychiatric practice in Texas. | Service evaluation/audit | Community mental health teams and outpatient services | Psychosis, bipolar, depression, anxiety, PTSD, other mental disorders | NA (description of service change) | Video call, Phone call, Text message/SMS/WhatsApp |
| Scharff et al. (2020) USA | Reporting the steps taken to shift to remote training and practice by a Psychological Services Centre. | Service evaluation/audit | Community based training clinic providing therapy | General population and individuals with anxiety and other mental disorders | NA (description of service change) | Video call |
| Schueller et al. (2019) USA | Review the use of digital mental health interventions in marginalised and underserved populations. | Review | No specific setting | NA | NA (review) | General telemental health |
| Sehlo et al. (2021) Egypt | Comparing patient views on F2F consultation and telepsychiatry in Egypt during the pandemic. | Quantitative cross-sectional study | Online psychological therapy service | Mixed primary care or psychology/psychotherapy service users | Service users (n=183) | Mixed F2F and telemental health |
| Severe et al. (2020) USA | Examining factors influencing patients’ decisions to accept or decline telepsychiatry and their choice of telemental health modality. | Quantitative cross-sectional study and qualitative survey | Community mental health teams and outpatient services | Mixed secondary mental health service users | Service users (n=44) | Video call, Phone call, Other (pause/postpone care) |
| Sheehan et al. (2020) UK | Reporting the experiences of staff working across different mental health services for people with intellectual and other developmental disabilities. | Quantitative cross-sectional study and qualitative survey | Not stated/unclear | Intellectual and developmental disabilities | Mental health professionals working with people with intellectual disabilities and/or developmental disabilities (n=648) | NA |
| Shklarski et al. (2021) USA | Assessing psychotherapists’ challenges and  adaptations to providing remote therapy from home during the Covid-19 pandemic. | Quantitative cross-sectional study and qualitative interview study | No specific setting/general population | NA | Mixed mental health professionals (n=92 in survey, n=19 in interviews) | General telemental health |
| Shore et al. (2014) USA | Describing the Home-Based Telemental program and examining the feasibility of providing online mental healthcare to veterans at home. | Quantitative cross-sectional study | Veteran medical centre | Depression, PTSD, psychosis and bipolar disorder, anxiety, other mental disorders | Veteran service users (n=38) | Video call |
| Simon et al. (2021) UK | Exploring the views of NHS commissioners and managers on internet-based therapies and their implementation. | Qualitative interview study | No specific setting | NA | Individuals working in the NHS (n=10) | Video call, Phone call |
| Simpson et al. (2001a) Canada | Evaluating the provision of routine psychiatric care to a rural community via video call. | Service evaluation/audit and primary research study: Quantitative cross-sectional study and qualitative interview and focus group study | Outpatient telepsychiatry consultations delivered from a psychiatric hospital to local general hospitals | Psychosis and bipolar, depression, dementia, other mental health disorders | Mental health clinic staff (n=13 in focus group), psychiatrists (n=3 in interviews) | Video call, Mixed F2F and telemental health |
| Simpson et al. (2001b) Canada | Reporting patient experiences of a routine telepsychiatry service in Alberta, Canada. | Quantitative cross-sectional study | Tertiary-care psychiatry service | Mixed secondary mental health service users and service users with depression | Service users (n=230 in surveys, n=31 in structured telephone interviews) | Video call |
| Smith and Gillon (2021) UK | Exploring therapists’ views and experiences of processes and efficacy of online counselling. | Qualitative interview study | No specific setting | NA | Counsellors and Psychotherapist or analyst (n=5) | Video call, Phone call |
| The British Psychological Society (2020a) UK | Providing guidance for psychologists working with  children and young people  using online video platforms. | Guidance document | No specific setting | NA | Not applicable (guidance document) | Video call |
| The British Psychological Society, (2020b) UK | Providing guidance for working therapeutically with  parents and their infants during  pregnancy and postpartum using remote delivery platforms. | Guidance document | No specific setting | Prenatal and postnatal mental health disorders | NA (guidance document) | Video call, |
| The Health Foundation (2020) UK | Evaluating “Connecting With Telehealth to Children in Hospital” (CWTCH), a NHS telepsychiatry project within Child  and Adolescent Mental Health Service (CAMHS). | Service evaluation/audit: Quantitative cross-sectional study and qualitative survey and interview study | Community mental health teams and outpatient services | NA | Patients, families, clinicians, wider public (n not reported) | Video call |
| The Mental Health Innovation Network (2020) USA | Providing guidance for supporting refugee mental health during COVID-19. | Webpage with relevant information | No specific setting | NA | Not applicable (guidance webpage) | Text messages |
| Uscher-Pines et al. (2020a) USA | Understanding how the shift in delivery modes has affected mental health care during the COVID-19 pandemic. | Qualitative interview study | Hospital outpatient clinic; Private practice; other setting | NA | Outpatient psychiatrists (n=20) | Video call, Phone call |
| Uscher-Pines et al. (2020b) USA | Describing how clinicians used telemedicine for Opiate Use Disorder in conjunction with in-person care during the early stages of the COVID-19 pandemic. | Qualitative interview study | Community mental health teams and outpatient services*;* Private hospital/clinic*;* General hospital/physical health service | Opioid use disorder | Clinicians (n=18) | Video call, Phone call |
| Vera San Juan et al. (2021) UK | Investigating the experiences of service users during the switch to telemental health care. | Qualitative interview study | Mixed mental health services (outpatient; crisis and emergency; inpatient; voluntary sector/non-profit; psychology/psychotherapy/counselling services; general hospital/physical health service ) | Mixed primary care or psychology/psychotherapy service users and mixed secondary mental health service users | Service users (n=44) | Video call, Phone call, Text message/SMS/WhatsApp |
| Watson et al. (2021) UK | Investigating which factors influence the uptake of remote therapy among individuals with psychosis. | Quantitative cross-sectional study | Psychology/psychotherapy/counselling service | Psychosis and associated emotional difficulties | Service users (n=51) | General telemental health |
| Whistance et al. (2020) UK | Evaluating the use and value of video consulting, including satisfaction, clinical suitability, and acceptability. | Service evaluation/audit: Quantitative cross-sectional study and qualitative survey | Mixed mental health settings (primary, secondary and community care) | Mixed primary care or psychology/psychotherapy service users and mixed secondary mental health service users | Mixed groups of mental health professionals (n=6,090), service users (n=4,311) | Video call |
| Williams et al. (2017) UK | Investigating the views of people who are homeless or insecurely housed on telemental health. | Quantitative cross-sectional study and qualitative focus group study and interview study | Local open access homeless and wellbeing daycentre | NA | Service users (N=64: n=51 interviewees, n=13 focus group members) | General telemental health |
| Wilson et al. (2021) UK | Exploring staff perceptions of the impact of the COVID-19 pandemic on mental health service delivery and outcomes for perinatal women. | Quantitative cross-sectional study and qualitative survey | Community mental health teams; Crisis and emergency mental health service*s;* inpatient mental health services; perinatal services | Perinatal mental health problems | Mixed clinical staff working with women in the perinatal period (n=363) | Video call, Phone call, Mixed F2F and telemental health |
| Wyler et al. (2021) Switzerland | Exploring how adult patients with ADHD and their therapists experienced different modalities of therapy during the COVID-19 pandemic. | Quantitative cross-sectional study and quantitative survey | Community mental health teams and outpatient services | ADHD | Staff and service users (n=60 therapist/service user dyads) | Video call, Phone call, Other (F2F with the therapist wearing a face mask) |
| Yellowlees et al. (2020) USA | Describing the process, challenges, and lessons learned from the rapid conversion of an outpatient psychiatric clinic to a virtual telepsychiatry clinic. | Commentary/editorial with data | General hospital/physical health service | NA | NA (description of service change) | Video call, Phone call |
| YoungMinds (2020) UK | Exploring experiences of children and young people of using digital technology. | Discussion (Submission to COVID Committee’s inquiry) | No specific setting | General population | NA | General telemental health |
| Zheng and Gray (2014) USA | Examining the feasibility of empirically supported treatments implemented via videoconferencing. | Case studies | Psychology Telehealth Clinic at the University of Wyoming | PTSD | Service users (n=2) | Video call |
